# Developing a vocabulary and ontology for modeling insect natural history data: example data, use cases, and competency questions

**DOI:** 10.3897/BDJ.7.e33303

**Published:** 2019-03-13

**Authors:** Brian J. Stucky, James P. Balhoff, Narayani Barve, Vijay Barve, Laura Brenskelle, Matthew H. Brush, Gregory A Dahlem, James D. J. Gilbert, Akito Y. Kawahara, Oliver Keller, Andrea Lucky, Peter J. Mayhew, David Plotkin, Katja C. Seltmann, Elijah Talamas, Gaurav Vaidya, Ramona Walls, Matt Yoder, Guanyang Zhang, Rob Guralnick

**Affiliations:** 1 Florida Museum of Natural History, University of Florida, Gainesville, FL, United States of America Florida Museum of Natural History, University of Florida Gainesville, FL United States of America; 2 Renaissance Computing Institute, University of North Carolina, Chapel Hill, NC, United States of America Renaissance Computing Institute, University of North Carolina Chapel Hill, NC United States of America; 3 Oregon Health and Science University, Portland, OR, United States of America Oregon Health and Science University Portland, OR United States of America; 4 Department of Biological Sciences, Northern Kentucky University, Highland Heights, KY, United States of America Department of Biological Sciences, Northern Kentucky University Highland Heights, KY United States of America; 5 Department of Biological and Marine Sciences, University of Hull, Hull, United Kingdom Department of Biological and Marine Sciences, University of Hull Hull United Kingdom; 6 Entomology and Nematology Department, University of Florida, Gainesville, FL, United States of America Entomology and Nematology Department, University of Florida Gainesville, FL United States of America; 7 Department of Biology, University of York, York, United Kingdom Department of Biology, University of York York United Kingdom; 8 Florida Department of Agriculture and Consumer Services, Gainesville, FL, United States of America Florida Department of Agriculture and Consumer Services Gainesville, FL United States of America; 9 Bio5 and CyVerse, University of Arizona, Tucson, AZ, United States of America Bio5 and CyVerse, University of Arizona Tucson, AZ United States of America; 10 Species File Group, Illinois Natural History Survey, University of Illinois, Champaign, IL, United States of America Species File Group, Illinois Natural History Survey, University of Illinois Champaign, IL United States of America

**Keywords:** insects, natural history, biodiversity informatics, ontology, data modeling

## Abstract

Insects are possibly the most taxonomically and ecologically diverse class of multicellular organisms on Earth. Consequently, they provide nearly unlimited opportunities to develop and test ecological and evolutionary hypotheses. Currently, however, large-scale studies of insect ecology, behavior, and trait evolution are impeded by the difficulty in obtaining and analyzing data derived from natural history observations of insects. These data are typically highly heterogeneous and widely scattered among many sources, which makes developing robust information systems to aggregate and disseminate them a significant challenge. As a step towards this goal, we report initial results of a new effort to develop a standardized vocabulary and ontology for insect natural history data. In particular, we describe a new database of representative insect natural history data derived from multiple sources (but focused on data from specimens in biological collections), an analysis of the abstract conceptual areas required for a comprehensive ontology of insect natural history data, and a database of use cases and competency questions to guide the development of data systems for insect natural history data. We also discuss data modeling and technology-related challenges that must be overcome to implement robust integration of insect natural history data.

## Introduction

Insects are possibly the most diverse class of multicellular organisms on Earth, not only in sheer number of species, but also in terms of ecological diversity (Grimaldi and Engel 2005, Larsen et al. 2017). For example, insects encompass just about every sort of trophic strategy known in animals, including herbivory, scavenging, predation, parasitism, and parasitoidism. In some cases, all of these strategies are found within a single taxonomic family (e.g., Disney 1994, Marshall 2012, Rainford and Mayhew 2015). Thus, insects present boundless opportunities to test hypotheses about the ecology and evolution of feeding behaviors, species interactions, habitat associations, and much more. Actually realizing this potential within a scientific study, however, is quite challenging because of the difficulty in obtaining, integrating, and analyzing suitable natural history data.

Currently, data about the natural history of insects are widely scattered among a multitude of sources, including labels on specimens in biological collections, specialized (and often obscure) publications, field notebooks, and taxon-specific databases. Thus, finding relevant natural history data for a given insect species can be a daunting task. Furthermore, insect natural history data are highly heterogeneous. For example, they commonly differ in observational methodology (e.g., observations in the field versus in the lab), observational detail (e.g., differences in temporal resolution or certainty of biotic associations), or in the terminology used by the observers. Aggregating these data so that they can be analyzed and disseminated efficiently, without information loss, is a major informatics challenge.

A critical step towards meeting this challenge is developing comprehensive standards to guide the design and implementation of data systems for aggregating insect natural history data. To support robust data integration, these standards need to include two major components: first, a well-defined vocabulary of natural history terms that is suitable for recording natural history observations across all insect taxa and, second, an ontology that provides computable semantics for the vocabulary so that computers can understand how the terms in the vocabulary relate to one another (ontologies are described in the next section). Such data standards can have a major impact on large-scale biodiversity science, as exemplified by the success of the "Darwin Core" vocabulary for aggregating and exchanging species occurrence data (Wieczorek et al. 2012).

Here, we report initial results of a new effort to develop a standardized vocabulary and ontology for insect natural history data, an effort that was initiated at a three-day workshop, held at the University of Florida from 1 May to 1 June 2018, that convened entomologists, computer scientists, and data modelers. Although work on a draft ontology is still in progress, in this short communication we describe several key results of our work so far that are likely to be of broader interest, including an analysis of high-level ontology concept areas, a conceptually comprehensive database of example insect natural history data, and a database of ontology use cases and ontology competency questions.

To make this work tractable, we have mostly focused on natural history information from specimens in collections, with taxonomic scope limited to the five mega-diverse insect orders (Hemiptera, Coleoptera, Diptera, Lepidoptera, Hymenoptera), which include the vast majority of insect species and ecological diversity (Grimaldi and Engel 2005). We also excluded natural history information inferred from fossil material. There is considerable overlap in content between insect natural history data from specimen labels and from other sources (e.g., literature), so much of our work will be easily adaptable to information about other insect orders or information from sources other than specimen labels. Looking even further ahead, we anticipate that an ontology for insect natural history data could eventually serve as a foundation for developing a broader ontology for natural history data that also includes other groups of animals.

Before turning to discussion of our vocabulary and ontology development efforts, we recognize that many readers might have little experience with ontologies, so we briefly introduce ontologies and why they are important for integrating natural history data.

## A (very) brief introduction to ontologies

**Figure 1.**
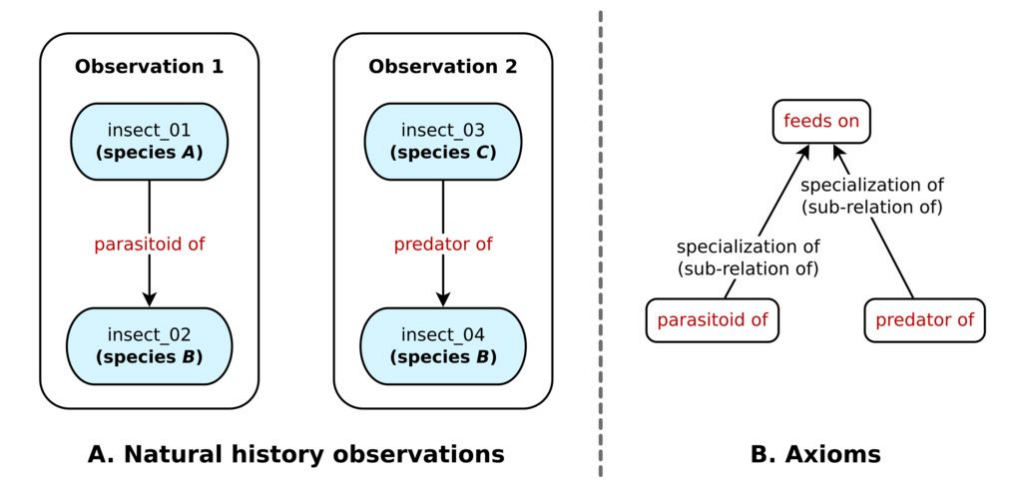
Example natural history observations **A** and ontology axioms **B**. The axioms allow a computer to understand, for example, that if insect_01 is a “parasitoid of” insect_02, it is also true that insect_01 “feeds on” insect_02.

An *ontology*, as the term is used in computer and information science, is an explicit, precise, machine-interpretable conceptualization of some knowledge domain. Although we do not have space in this manuscript to provide a detailed introduction to ontologies, we will try to provide some intuition by way of a simple example. Suppose we have two natural history observations: observation 1 asserts that an individual of species *A* was a parasitoid of an individual of species *B* and observation 2 asserts that an individual of species *C* was a predator of species *B* (Fig. 1). Now, suppose we have a database that includes these two observations (and potentially many more), and we wish to query the database to find all of the species that are known to use species *B* as a food source.

Given observations 1 and 2, a human biologist can easily infer that species *A* and *C* are both known to feed on species *B*, but a computer does not automatically understand that “parasitoid of” and “predator of” both imply trophic relationships. With an ontology, we can provide formal logic statements, called *axioms*, that allow a computer to make this inference. To continue with the example, we could write axioms that assert that the relationships “parasitoid of” and “predator of” are both special cases of a more general relationship called “feeds on” Fig. 1. Armed with this information, a computer could directly answer our question about which species use species *B* as a food source.

With only two observations and a few vocabulary terms, this might seem like a trivial accomplishment, but when we have hundreds, thousands, or even millions of heterogeneous natural history observations, with hundreds of logical relationships among the terms in a large vocabulary, ontologies make it possible to automate complex data integration and querying tasks that would be practically impossible for a human. Thus, ontologies are critical to any effort to develop robust systems for aggregating insect natural history data. Furthermore, although this brief discussion has focused on the value of ontologies for data aggregators and users, ontologies are also beneficial for data creators and providers because they provide a standardized vocabulary that, once adopted, makes an individual's or organization's data immediately interoperable with similar data from other sources. This, in turn, makes the data more likely to be used (and cited) by other researchers. For readers who wish to learn more about data modeling with ontologies, Allemang and Hendler (2011) provide a good introduction.

## Development tasks, methods, and outcomes

We now return to discussion of the ontology design and development work initiated at the workshop, which has been organized around four major tasks: 1) assembly of example data; 2) analysis of example data and ontology scoping; 3) high-level ontology design and concept identification; and 4) identifying use cases (and users) and authoring ontology competency questions. We briefly describe each of these tasks and present the results of our work so far.

### Assembly of example data

Insect natural history is an extremely broad domain, which means that identifying an appropriate scope for a new data vocabulary and ontology is not a simple task. Our approach to this problem was to assemble example natural history data, drawn from real data sources, for each of the five major insect orders. This served two purposes. First, examining a well-drawn set of example data is a practical method for delimiting the scope of a new vocabulary and ontology, and second, a good example dataset also provides valuable test cases for use during vocabulary and ontology development.

To generate the example dataset, we worked in five small groups. Each group was assigned one of of the five major insect orders, and we ensured that each group included at least one entomologist with expertise in the assigned order. Then, each group gathered example natural history data for their insect order, with the goal of compiling a concise dataset that represented the various kinds of natural history information recorded on specimen labels for each major insect order. We attempted to capture both the breadth of biological information and the range of observational detail found in label data. Although we focused on information from insect specimen labels, we also included some data from literature sources and online databases such as iNaturalist (https://www.inaturalist.org) and GloBI (Poelen et al. 2014). For data from specimen labels, we used specimens and labels with digital images available on iDigBio (Page et al. 2015) whenever possible. Example data we gathered at the workshop were supplemented by additional example data that a few participants gathered both prior to and after the workshop.

Our final dataset includes 189 natural history observations covering a wide range of concepts and observation types (see next section). We expect that this dataset will have value to other researchers as well, so we have included it with this manuscript as two supplemental files, with one file formatted as a PDF document (Suppl. material 1) and one file in tabular comma-separated values (CSV) format (Suppl. material 2). Both of these files are also available in a public git repository hosted on GitLab which provides the example data in other formats, too, including styled HTML, Markdown, and a SQLite database (https://gitlab.com/stuckyb/inhd_ontology/tree/master/example_data).

### Analysis of example data and ontology scoping

After assembling the example data, we used them to delimit the high-level scope of the new vocabulary and ontology. Again working in small groups, we analyzed the kinds of information contained in the example data, with each group focusing on one of the five major insect orders. For each order, we summarized the kinds of biological information that were observed (e.g., various multi-organism interactions, developmental data) and the ways in which the information was recorded (e.g., qualitative or quantitative). Then, we reconvened as a large group, each small group reported their findings, and we synthesized the results to arrive at a set of 10 high-level conceptual areas required for the final ontology (Table 1).

Together, these conceptual areas cover virtually all of the kinds of information contained in the example data we assembled, and we therefore propose that an ontology that provides suitable coverage of all 10 of these areas will be sufficient for modeling nearly all insect natural history data from specimen labels as well as a substantial proportion of insect natural history data from other sources, including literature-based data. This conclusion is dependent, of course, on the extent to which our example data capture the conceptual breadth and depth of all available insect natural history information. Although we were not able to formally evaluate this, given the collective entomological expertise of the workshop participants (many of whom have years of experience examining specimens and labels from entomology collections around the world) and the effort spent compiling example data, we are confident that we at least came close to achieving this goal for natural history data from insect specimen labels.

We also note that several of these conceptual areas overlap with the domains of extant ontologies, and in Table 2, we list the ontologies that are most relevant to each conceptual area. To ensure broad compatibility, reusability, and extensibility, we plan to use existing ontological resources wherever possible and contribute (or suggest) new entities for extant ontologies, when appropriate.

### High-level ontology design and concept identification

Of the 10 conceptual areas we identified (Table 1), we determined that observations and observing processes, relationships and interactions, and positional (spatial) information were the most critical for developing an immediately useful vocabulary and ontology. Our decision to prioritize these areas was based on three considerations. First, observations and observing processes underlie *all* insect natural history data and encompass the crucial "who", "when", and "where" information about such data. Second, relationships and interactions are of broad scientific interest because they provide the raw ecological information needed for a wide variety of research topics (e.g., understanding trophic relationships, discovering potential disease vectors, or predicting the consequences of ecosystem changes). Third, we found that positional or spatial information is often included on specimen labels and in literature-based natural history observations, and we therefore concluded that even a minimal data standard should be able to capture such information. After prioritizing these three conceptual areas, we again worked in groups to begin sketching out data models (Simsion 2007, Simsion and Witt 2005) and ontology design patterns (Gangemi 2005) for all three areas and to identify the entities (concepts) to include in each conceptual area.

This initial design work revealed several critical data modeling challenges, the thorniest of which is the problem of recording metadata about natural history observations that include interactions between organisms. Such observations are common in natural history data and include, for example, observations about feeding relationships, parasite/host relationships, courtship, and many more. As with any other natural history observation, it is important to be able to record metadata about interaction observations, such as who made the observations, when they occurred, and so on. Without plunging into too much technical detail, the central problem is that the technology most often used for implementing ontology-enabled data, the Resource Description Framework (RDF, Miller 2005), currently has poor support for expressing metadata about interactions or relationships (Hartig 2017). A number of workarounds have been proposed (e.g., Hartig 2017, Nguyen et al. 2014, Hernández et al. 2015), but most of them have undesirable consequences, such as artificially increasing database size, complicating query statements, or slowing query response times (Hartig 2017, Hernández et al. 2015). Our work on this is ongoing, and we are actively investigating several different implementation strategies.

A second important data modeling problem is the challenge of accurately capturing information about *what* organisms were observed, which means dealing with the myriad difficulties posed by the use of taxonomic names (Zermoglio et al. 2016, Hardisty et al. 2013, Remsen 2016, Pyle 2016, Patterson et al. 2016). These issues are especially severe when dealing with data about insects, simply because insects are so extraordinarily diverse: many species remain undescribed and specimens in collections are often not identified to species (indeed, for some diverse insect families, the *majority* of specimens in a collection might not be identified to species). Relatively frequent – and sometimes dramatic – taxonomic changes mean that the names used in publications and labels can quickly become inaccurate or obsolete. These issues are certainly not unique to insect natural history data, and we have not attempted to add to the substantial work already done in this area (e.g., Franz et al. 2017, Franz and Peet 2009, Hardisty et al. 2013, Pyle 2016). For now, though, taxonomic integration remains a major challenge for virtually all biodiversity-related data aggregation efforts, and insect natural history data are no exception.

### Identifying use cases and authoring ontology competency questions

The last major task of our preliminary design and development work was drafting detailed ontology competency questions and identifying potential users and user cases. Ontology competency questions (OCQs, Grüninger and Fox 1995, Ren et al. 2014) provide a means for testing an ontology by providing specific queries that an ontology (along with an associated database) ought to be able to answer. In other words, OCQs specify how an ontology will be used to ask questions of real data. Thus, writing OCQs goes hand-in-hand with determining an ontology's users and use cases. To give a couple of examples, OCQs for an ontology of insect natural history data might include, “On what substrates does species *A* lay its eggs?” or “Has species *B* been collected at artificial lights?”

To identify use cases and develop OCQs, we divided into three groups on the last day of the workshop, with each group working independently and recording their results. After the workshop, one of us (BJS) synthesied the results of each group’s efforts into a single, comprehensive set of use cases and OCQs. The use cases we identified cover seven main user groups or domains:

1. Entomology (e.g., insect collecting and rearing, forensic entomology).
2. Taxonomy and systematics (e.g., field guides, systematic revisions).
3. Ecology and evolutionary biology (e.g., disease ecology, comparative studies).
4. Conservation biology and natural resource management (e.g., ecological restoration, environmental monitoring).
5. Agriculture and forestry (e.g., identifying potential pest insects, identifying potential disease vectors).
6. Education (e.g., classroom education, public outreach).
7. The general public (e.g., researching garden pests and control agents, hobby insect collecting).

The full sets of use cases and OCQs are too large to report in the main text, so we instead provide them in Suppl. material 3. The use cases and OCQs are also available in a public git repository on GitLab, which includes a SQLite database of use cases and OCQs along with example queries (https://gitlab.com/stuckyb/inhd_ontology/tree/master/OCQs).

## Conclusions

With the work and results reported in this paper, we have laid a foundation for ongoing efforts to design, develop, and implement a robust vocabulary and ontology for modeling insect natural history data. Our next immediate goals are to identify the best solution for dealing with the problem of interactions metadata, discussed above, and to produce and release a draft ontology implementation for public review. We welcome additional participants in these efforts; readers who would like to be involved should contact the corresponding author (BJS). In the meantime, we hope that the foundational work reported in this paper, including the comprehensive example dataset and OCQs, will prove useful to other researchers interested in the informatics challenges surrounding insect natural history data.

## Supplementary Material

Supplementary material 1 Example insect natural history data (PDF document) Data type: natural history
File: oo_258716.pdf Brian Stucky, James Balhoff, Narayani Barve, Vijay Barve, Laura Brenskelle, Matthew H. Brush, Gregory Dahlem, James Gilbert, Akito Kawahara, Oliver Keller, Andrea Lucky, Peter Mayhew, David Plotkin, Katja Seltmann, Elijah Talamas, Gaurav Vaidya, Ramona Walls, Matt Yoder, Guanyang Zhang, Rob Guralnick

Supplementary material 2 Example insect natural history data (CSV file) Data type: natural history
File: oo_258720.csv Brian Stucky, James Balhoff, Narayani Barve, Vijay Barve, Laura Brenskelle, Matthew H. Brush, Gregory Dahlem, James Gilbert, Akito Kawahara, Oliver Keller, Andrea Lucky, Peter Mayhew, David Plotkin, Katja Seltmann, Elijah Talamas, Gaurav Vaidya, Ramona Walls, Matt Yoder, Guanyang Zhang, Rob Guralnick

Supplementary material 3 Ontology competency questions, user domains or groups, and example use cases Data type: tables
File: oo_259027.pdf Brian Stucky, James Balhoff, Narayani Barve, Vijay Barve, Laura Brenskelle, Matthew H. Brush, Gregory Dahlem, James Gilbert, Akito Kawahara, Oliver Keller, Andrea Lucky, Peter Mayhew, David Plotkin, Katja Seltmann, Elijah Talamas, Gaurav Vaidya, Ramona Walls, Matt Yoder, Guanyang Zhang, Rob Guralnick

## Figures and Tables

**Table 1.** Ten top-level conceptual areas required for a comprehensive ontology of insect natural history data. "Relevant extant ontologies" are existing ontologies that provide at least partial coverage of the concepts in a given conceptual area. All of the ontologies mentioned here are part of the Open Biological and Biomedical Ontology (OBO) Foundry (Smith et al. 2007), a collection of interoperable ontologies that has been widely adopted in the biological sciences. Ontology references: **1.** Walls et al. (2018), **2.** Walls et al. (2014), **3.** Gene Ontology Consortium (2004), **4.** Ashburner et al. (2000), **5.** Smith et al. (2005), **6.** Gkoutos et al. (2012), **7.** Mungall et al. (2012), **8.** Buttigieg et al. (2016), **9.** Buttigieg et al. (2013), **10.** Ashburner and Schriml (2013), **11.** Dahdul et al. (2014).

**Conceptual area**	**Description**	**Relevant extant ontologies**
Observations and observing processes	Observations of insect natural history and the processes that generate them, including information about the observers (whether human or machine) and where and when observations are made.	Biological Collections Ontology [1,2]
Relationships and interactions	Behaviors that involve interactions among organisms. Includes pairwise interactions (e.g., mating or herbivory) and multi-way interactions (e.g., cooperative colony defense or ants defending aphids from a potential predator).	Gene Ontology [3,4], Relations Ontology [5]
Single-organism behaviors	Behaviors that do not necessarily involve interactions with other organisms (e.g., perching or locomotion).	Neurobehavior Ontology [6]
Ontogeny	Developmental information (e.g., instar number or length of larval stage).	Gene Ontology [3,4], Uberon [7]
Organism products and traces	Non-living objects or artifacts generated by insects (e.g., nests or leaf mines).	
Habitat, locality, and substrates	The physical context in which an organism is found, at all scales (e.g., a geopolitical boundary or a specific microhabitat).	Environment Ontology [8,9], GAZ [10]
Positional and spatial information	Information about the location of an organism relative to some other object or reference point (e.g., underneath the bark of a log, the south side of a rock).	Biological Spatial Ontology [11], Relations Ontology [5]
Weather and climate	Information about weather conditions or climate (e.g., momentary or long-term observations of temperature or precipitation) at any spatial scale.	
Collecting methods	The methods used to obtain specimens or individuals for observation (e.g., sweep netting or pitfall trapping) and information about how those methods are implemented.	Biological Collections Ontology [1,2]
Curation	Information about how specimens or other artifacts are managed (e.g., where they are housed and how they are preserved).	Biological Collections Ontology [1,2]

## References

[B4993980] Allemang Dean, Hendler J. A. (2011). Semantic Web for the Working Ontologist: Effective Modeling in Rdfs and Owl.

[B5011257] Ashburner Michael, Ball Catherine A., Blake Judith A., Botstein David, Butler Heather, Cherry J. Michael, Davis Allan P., Dolinski Kara, Dwight Selina S., Eppig Janan T., Harris Midori A., Hill David P., Issel-Tarver Laurie, Kasarskis Andrew, Lewis Suzanna, Matese John C., Richardson Joel E., Ringwald Martin, Rubin Gerald M., Sherlock Gavin (2000). Gene Ontology: tool for the unification of biology. Nature Genetics.

[B5011960] Ashburner Michael, Schriml Lynn (2013). GAZ: An open source gazetteer constructed on ontological principles. http://environmentontology.github.io/gaz/.

[B5011283] Buttigieg Pier, Morrison Norman, Smith Barry, Mungall Christopher J, Lewis Suzanna E, Consortium the ENVO (2013). The environment ontology: contextualising biological and biomedical entities. Journal of Biomedical Semantics.

[B4993989] Buttigieg Pier Luigi, Pafilis Evangelos, Lewis Suzanna E., Schildhauer Mark P., Walls Ramona L., Mungall Christopher J. (2016). The environment ontology in 2016: Bridging domains with increased scope, semantic density, and interoperation. Journal of Biomedical Semantics.

[B5011941] Dahdul Wasila M, Cui Hong, Mabee Paula M, Mungall Christopher J, Osumi-Sutherland David, Walls Ramona L, Haendel Melissa A (2014). Nose to tail, roots to shoots: spatial descriptors for phenotypic diversity in the Biological Spatial Ontology. Journal of Biomedical Semantics.

[B4993870] Disney R. H. L. (1994). Scuttle Flies: The Phoridae.

[B5011210] Franz Nico M., Zhang Chao, Lee Joohyung (2017). A logic approach to modelling nomenclatural change. Cladistics.

[B5011200] Franz N. M., Peet R. K. (2009). Perspectives: Towards a language for mapping relationships among taxonomic concepts. Systematics and Biodiversity.

[B4994066] Gangemi Aldo (2005). Ontology design patterns for semantic web content. The Semantic Web – ISWC 2005. Lecture Notes in Computer Science.

[B5011247] Gene Ontology Consortium (2004). The Gene Ontology (GO) database and informatics resource. Nucleic Acids Research.

[B5011220] Gkoutos Georgios V., Schofield Paul N., Hoehndorf Robert, Chesler Elissa J., Haendel Melissa A. (2012). The Neurobehavior Ontology. International Review of Neurobiology: Bioinformatics of Behavior: Part 1.

[B4993861] Grimaldi D. A., Engel M. S. (2005). Evolution of the Insects.

[B4993888] Grüninger Michael, Fox M. S., Rolstadås A. (1995). The role of competency questions in enterprise engineering. Benchmarking — Theory and Practice. IFIP Advances in Information and Communication Technology.

[B5011160] Hardisty Alex, Roberts Dave, community The biodiversity informatics (2013). A decadal view of biodiversity informatics: challenges and priorities. BMC Ecology.

[B4994056] Hartig Olaf (2017). Foundations of RDF and SPARQL: An alternative approach to statement-level metadata in RDF. CEUR Workshop Proceedings.

[B4994036] Hernández Daniel, Hogan Aidan, Krötzsch Markus (2015). Reifying rdf: what works well with Wikidata?. CEUR Workshop Proceedings.

[B4993902] Larsen B. B., Miller E. C., Rhodes M. K., Wiens J. J. (2017). Inordinate fondness multiplied and redistributed: The number of species on Earth and the new pie of life. The Quarterly Review of Biology.

[B4993879] Marshall S. A. (2012). Flies: The Natural History & Diversity of Diptera.

[B4993912] Miller Eric (2005). An introduction to the Resource Description Framework. Bulletin of the American Society for Information Science and Technology.

[B5011236] Mungall Christopher J, Torniai Carlo, Gkoutos Georgios V, Lewis Suzanna E, Haendel Melissa A (2012). Uberon, an integrative multi-species anatomy ontology. Genome Biology.

[B4994046] Nguyen Vinh, Bodenreider Olivier, Sheth Amit (2014). Don't like RDF reification?: Making statements about statements using singleton property.

[B4993922] Page Lawrence M., MacFadden Bruce J., Fortes Jose A., Soltis Pamela S., Riccardi Greg (2015). Digitization of biodiversity collections reveals biggest data on biodiversity. BioScience.

[B5011190] Patterson David, Mozzherin Dmitry, Shorthouse David, Thessen Anne (2016). Challenges with using names to link digital biodiversity information. Biodiversity Data Journal.

[B4993933] Poelen Jorrit H., Simons James D., Mungall Chris J. (2014). Global biotic interactions: An open infrastructure to share and analyze species-interaction datasets. Ecological Informatics.

[B5011180] Pyle Richard (2016). Towards a Global Names Architecture: The future of indexing scientific names. ZooKeys.

[B4993943] Rainford J. L., Mayhew P. J. (2015). Diet evolution and clade richness in Hexapoda: A phylogenetic study of higher taxa. The American Naturalist.

[B5011150] Remsen David (2016). The use and limits of scientific names in biological informatics. ZooKeys.

[B4993953] Ren Y, Parvizi A, Mellish C, Pan J. Z., Deemter K van, Stevens R (2014). Towards competency question-driven ontology authoring. The Semantic Web: Trends and Challenges. ESWC 2014. Lecture Notes in Computer Science.

[B4994027] Simsion Graeme C. (2007). Data Modeling: Theory and Practice.

[B4994018] Simsion G. C., Witt G. C. (2005). Data Modeling Essentials.

[B4993814] Smith Barry, Ceusters Werner, Klagges Bert, Köhler Jacob, Kumar Anand, Lomax Jane, Mungall Chris, Neuhaus Fabian, Rector A. L., Rosse Cornelius (2005). Relations in biomedical ontologies. Genome Biology.

[B5011295] Smith Barry, Ashburner Michael, Rosse Cornelius, Bard Jonathan, Bug William, Ceusters Werner, Goldberg Louis J, Eilbeck Karen, Ireland Amelia, Consortium The OBI, Mungall Christopher J, Leontis Neocles, Rocca-Serra Philippe, Ruttenberg Alan, Sansone Susanna-Assunta, Scheuermann Richard H, Shah Nigam, Whetzel Patricia L, Lewis Suzanna (2007). The OBO Foundry: coordinated evolution of ontologies to support biomedical data integration. Nature Biotechnology.

[B4993830] Walls R. L., Deck John, Guralnick Robert, Baskauf Steve, Beaman Reed, Blum Stanley, Bowers Shawn, Buttigieg P. L., Davies Neil, Endresen Dag, Gandolfo M. A., Hanner Robert, Janning Alyssa, Krishtalka Leonard, Matsunaga Andréa, Midford Peter, Morrison Norman, Tuama É. Ó., Schildhauer Mark, Smith Barry, Stucky B. J., Thomer Andrea, Wieczorek John, Whitacre Jamie, Wooley John (2014). Semantics in support of biodiversity knowledge discovery: an introduction to the Biological Collections Ontology and related ontologies. PLOS One.

[B4994001] Walls R. L., Buttigieg P. L., Deck John, Guralnick Rob, Wieczorek John (2018). Integrating and managing biodiversity data with the Biocollections Ontology. Application of Semantic Technology in Biodiversity Science.

[B5009649] Wieczorek John, Bloom David, Guralnick Robert, Blum Stan, Döring Markus, Giovanni Renato, Robertson Tim, Vieglais David (2012). Darwin Core: An evolving community-developed biodiversity data standard. PLoS ONE.

[B5011170] Zermoglio Paula F., Guralnick Robert P., Wieczorek John R. (2016). A Standardized Reference Data Set for Vertebrate Taxon Name Resolution. PLOS ONE.

