# Supplementary material for: Developing a vocabulary and ontology for modeling insect natural history data: example data, use cases, and competency questions
Source: Biodivers Data J. 2019 Mar 13;7:e33303. doi: 10.3897/BDJ.7.e33303 (PMC6426826; doi:10.3897/BDJ.7.e33303)
Supplement: Supplementary material 1 — Example insect natural history data (PDF document) [file bdj-07-e33303-s001.pdf]

Example Insect Natural History Data

These data were assembled by participants of a workshop held at the University of Florida from May 30 to June 1 of 2018. The data cover all five major insect orders (Coleoptera, Diptera, Hemiptera, Hymenoptera, Lepidoptera) and represent most of the various kinds of natural history information found on insect specimen labels. The data also include representative natural history information from literature sources and online databases. For more information about how these data were assembled and why, see Stucky et al. (2019) __________. Except for works in the public domain, data use licenses are as specified by the original data owners.

Coleoptera

Example 1

Taxonomy: Coleoptera: Buprestidae: *Acmaeodera* sp.

Record type: database

Life stage(s): adult

Source: iNaturalist

Record URL: https://www.inaturalist.org/observations/12840335

Comments and relevant content:

"Feeding on wildflowers in an open meadow in the midlands of South Carolina."

Example 2

Taxonomy: Coleoptera: Cerambycidae

Record type: literature

Source: Paro et al. (2011)

Relevant text:

"Table 1. Association between girdled and available host-plants (listed alphabetically) and Onciderini beetles in Serra do Japi from 2002 to 2006." The table gives the percentages of each plant species that were girdled along with associated beetle species.

Example 3

Taxonomy: Coleoptera: Cerambycidae: *Rhaesus serricollis*

Record type: literature

Source: Sama et al. (2010)

Relevant text:

"Host plants: Polyphagous on deciduous trees like Platanus (Platanaceae), Ficus (Moraceae), Quercus ithaburensis (Bytinski -Salz, 1956: 210); Q. calliprinos (Bytinski-Salz & Sternlicht, 1967); Platanus orientalis L., Populus (Halperin & Holzschuh, 1993). "

Example 4

Taxonomy: Coleoptera: Chrysomelidae: *Labidomera clivicollis*

Record type: database

Life stage(s): adult

Source: iNaturalist

Record URL: https://www.inaturalist.org/observations/12887649

Comments and relevant content:

"Seen feeding on Asclepias tuberosa"

Example 5

Taxonomy: Coleoptera: Coccinellidae: *Harmonia axyridis*

Record type: database

Life stage(s): larva

Source: iNaturalist

Record URL: https://www.inaturalist.org/observations/11659450

Comments and relevant content:

"Ladybeetle larva feeding on aphid on coral honeysuckle"

Example 6

Taxonomy: Coleoptera: Curculionidae: *Cnestus mutilatus*

Record type: literature

Life stage(s): adult

Source: Carlton and Bayless (2011)

Relevant text:

"They [the photographs] depict a plastic gasoline container used for home fuel storage with numerous holes and embedded specimens of a xyleborine scolytine. Photographs of the scolytines allowed us to identify the causal agents as C. mutilatus females."

"One contained gasoline/oil 2-cycle mix. The other two contained gasoline only. The gasoline was purchased at a local station and had a 10% ethanol component. An 8-L container had 63 borings, a 19-L container had 48, and a 23-L container had 157. Several holes had been bored completely through so that when the containers were lifted to pour, the gasoline leaked from the holes. Numerous holes also contained dead females of C. mutilatus (Fig. 1). Surrounding habitat was open pasture and lawn with scattered large live oak trees."

"Our assessment is that the containers were leaching volatiles (e.g., ethanol, a known attractant of scolytines [Ranger et al. 2010]) that stimulated boring by female C. mutilatus."

Example 7

Taxonomy: Coleoptera: Curculionidae: *Dendroctonus ponderosae*

Record type: database

Source: Atkinson (no date)

Record URL: http://barkbeetles.info/individual_record.php?lookUp=484&full_name=Dendroctonus%20ponderosae%20%20Hopkins&series_code=125081

Comments and relevant content:

Asserts that D. ponderosae has a host plant association with Pinus contorta. Includes collector information, so this is evidently based on one or more specimens.

Example 8

Taxonomy: Coleoptera: Helophoridae: *Helophorus oblongus*

Record type: specimen

Source: Webster and Sweeney (2016)

Relevant label text:

"Black spruce forest, flooded semi-permanent sedge marsh [pond]"

Example 9

Taxonomy: Coleoptera: Hydrophilidae: *Berosus fraternus*

Record type: specimen

Source: Webster and Sweeney (2016)

Relevant label text:

"Mixed forest, gravel bottomed pool near roadside"

"Silver maple forest, u.v. light trap near marsh"

"Mixed forest, small pool on forest trail"

Example 10

Taxonomy: Coleoptera: Lampyridae: *Ellychnia corrusca*

Record type: literature

Life stage(s): adult, larva

Source: Faust (2012)

Relevant text:

"These fireflies, widespread but less abundant than other local firefly species, are found in loose colonies on the margins or within the secondary, mixed hardwood forests of East TN, which are predominantly Appalachian oak-hickory type (Fig. 3)."

"The studies for the two firefly species took place over four seasons (2008–11). Outside the firefly season from May–January, monthly site visits recorded sightings of adults or larvae on trees or the ground. The sites were visited weekly from the first appearance of E. corrusca, in Jan or Feb through early May (last sightings for P.borealis). For both firefly species, site visits recorded the number of individuals found on each of their respective colony trees. For each individual, the following was also recorded: height from the ground; orientation in relation to:

1) aspect on tree (i.e., amount of solar radiation received, where the south-facing side is warmest),

2) the vertical line of the tree trunk,

3) other individuals; activity level (slow or fast-moving); sex (of adults for E. corrusca, when possible and at the pupal/adult stage for P. borealis). Aspects of behaviour during primary life events were recorded (quiescence, escape strategies, dispersal, mating and oviposition for E. corrusca; escape strategies, pupation, pupal-guarding, eclosion, courtship and mating for P. borealis). Mortality and likely causes were recorded, and also the presence of other insects resembling these fireflies."

"One glowing larva, found in September, white and pink from recent molting, had darkened by the next morning (Fig. 5). It measured 15–17 mm long and 3.5 mm wide and readily ate small chopped live worms 6 times in 21 days. It used its pygypodia (caudal tail organ) for grooming and to pry open a fresh water snail operculum. It did not attack a more fragile Photinus sp. larva kept in the same enclosure. They often fed together, though the Photinus larva deferred to it. After six weeks, it died from unknown causes."

"From late October to early February, few to no adults or larvae were evident either on the ground or tree trunks. Adults were first sighted at one or more of the sites on 14, 10 and 18 February in 2008, 2009 and 2010, respectively, and on 20 January in 2011. At Knox (29 trees) in 2010 (Fig. 6), no adults were seen until 20 February when 44 were found. The next day's total was 104. Smaller groups emerged over the final week in February, usually on clear sunny days, with a late group of seven appearing on 29 March bringing to a total of 217 adults for that site and 299 for all sites."

"When emerging at temperatures below 5°C, often with snow-covered ground, the adults moved slowly. Mean height up the tree trunks increased daily from 66 cm (on 18 February) to 88 cm, 152 cm, and 170 cm on Feb 21 (range 15-152 cm, 22-243 cm, 96-307 cm, 0-320 cm respectively) at the Morgan and Knox sites with temperatures at appearance ranging from 0-16°C."

"Dispersal, recognised by the reappearance of fireflies in flight or active on both colony and non-colony trees, occurred in mid-March at air temperatures above 15°C. First observed flight in 2010 and 2011 was on 8 March at 20°C and 17 March at 23°C, respectively. On very sunny days where south-facing trunk temperatures exceeded 25°C, adultswere often seen on the north sides of the still-leafless trees where temperatures were 5-10°C cooler."

"When observed too closely or feeling threatened, their two primary escape strategies were actively crawling up the trunk or dropping to the ground on their backs, becoming well-camouflaged in the leaf litter."

"They exuded white beads of reflexive bleeding from the elytral margins when severely threatened."

"Predation was recorded early (28 February) in the season for 2010 and 2011 and parasitism late (22 April) in the 2011 season. In 2010, two of four males taking shelter in a hole at the base of a Quercus alba tree were preyed on and in 2011, three unmarked elytra were found at the base of colony trees in thick leaf litter. Bite marks and/or tears on elytral remnants were consistent with rodent or bird predation. In 2011, 32 phorid larvae (Brown 1994) that emerged from a female that died shortly after, were later identified (Brown, pers. comm.) as Apocephalus antennatus Malloch (Diptera; Fig. 8). Beetles and moths from several families with mimic-like colouration and patterns were often found on or near the firefly colony trees (Fig. 9)."

Example 11

Taxonomy: Coleoptera: Lampyridae: *Pleotomodes needhami*

Record type: literature

Life stage(s): larva

Source: Sivinski et al. (1998)

Relevant text:

"These insects were found throughout the colonies during nocturnal observations. In two [ant] nests with fungal chambers there were 19 instances of larvae in the fungal chamber, 41 in tunnels, and 14 on the surface. The latter were often feeding on snails. In a third nest, without a fungal chamber, there were 18 observations of larvae in tunnels and 7 of larvae on the surface. Although larvae failed to develop during the survival of the colony, they fed, moved and even crawled into crevices in the fungal masses."

"There were no obvious interactions between the ants and firefly larvae. Larvae were generally motionless in the presence of ants and ants often squeezed past beetles in partially blocked tunnels. We saw no evidence of trophallaxis."

Example 12

Taxonomy: Coleoptera: Scarabaeidae: *Cyclocephala atricapilla*

Record type: literature

Life stage(s): adult

Source: Gottsberger (1989)

Relevant text:

"In the first evening, during the female phase of the flowers, the beetles began to arrive shortly before 20:00, which coincides with the initiation of the accuentuated flower heating. However, it was evident that at the temperature peak the number of beetles arriving notably increased. Afterwards, when the flower started to cool down, the approach of the new beetles diinished and at about 22:00 ceased. The beetles arrived in a zig-zag-like flight, landed at the outer side of the flower and crawled between the imbricate inner petals into the pollination chamber."

Example 13

Taxonomy: Coleoptera: Silphidae: *Thanatophilus truncatus*

Record type: specimen

Life stage(s): adult

Source: iDigBio

Record URL: https://www.idigbio.org/portal/records/6539d542-830d-4e29-b2f5-c03b8ff60258

Label image: http://storage.idigbio.org/uaic/scan/UAIC1075/UAIC_1075160_labels_dorsal_lg.jpg

Relevant label text:

"ex: golden eagle carcass"

Diptera

Example 14

Taxonomy: Diptera: Agromyzidae: *Chromatomyia horticola*

Record type: specimen

Life stage(s): adult

Source: iDigBio

Record URL: https://www.idigbio.org/portal/records/da207359-628f-49f2-87a4-aa6020059abe

Label image: http://www.nhm.ac.uk/services/media-store/asset/f03cce1bd4d544665f05eaa7e0f373b40934432c/contents/preview

Relevant label text:

"reared from leaf mine"

Example 15

Taxonomy: Diptera: Asilidae

Record type: specimen

Life stage(s): larva

Source: iDigBio

Record URL: https://www.idigbio.org/portal/records/f21d9e25-ccb6-493e-81fc-c3dfeafd95de

Label image: https://invertnet.org/imagestor/vials/larges/2014/11/13/11211-0.jpg

Relevant label text:

"ex: in wood – under bark of large elm log (not in contact w/ soil)"

Example 16

Taxonomy: Diptera: Asilidae

Record type: specimen

Life stage(s): larva

Source: iDigBio

Record URL: https://www.idigbio.org/portal/records/f21d9e25-ccb6-493e-81fc-c3dfeafd95de

Label image: https://invertnet.org/imagestor/vials/larges/2014/11/13/11211-0.jpg

Relevant label text:

"in decayed wood"

Example 17

Taxonomy: Diptera: Asilidae: *Choerades gilva*

Record type: photograph

Life stage(s): adult

Source: Wikimedia Commons

Photograph: https://commons.wikimedia.org/wiki/File:Choerades_gilva.jpg

Description:

Photograph of robber fly with prey, found by searching the category "Asilidae with prey" on Wikimedia Commons. Both the robber fly and prey have a taxonomic identification (not necessarily to species).

Example 18

Taxonomy: Diptera: Asilidae: *Choerades* sp.

Record type: photograph

Life stage(s): adult

Source: Wikimedia Commons

Photograph: https://commons.wikimedia.org/wiki/File:Asilidae_-_Laphria_sp._preys_Polydrusus_sp..JPG

Description:

Photograph of robber fly with prey, found by searching the category "Asilidae with prey" on Wikimedia Commons. Both the robber fly and prey have a taxonomic identification (not necessarily to species).

Example 19

Taxonomy: Diptera: Asilidae: *Dioctria linearis*

Record type: photograph

Life stage(s): adult

Source: Wikimedia Commons

Photograph: https://commons.wikimedia.org/wiki/File:Dioctria_linearis_-_side_(aka).jpg

Description:

Photograph of robber fly with prey, found by searching the category "Asilidae with prey" on Wikimedia Commons. Both the robber fly and prey have a taxonomic identification (not necessarily to species).

Example 20

Taxonomy: Diptera: Asilidae: *Dysmachus fuscipennis*

Record type: photograph

Life stage(s): adult

Source: Wikimedia Commons

Photograph: https://commons.wikimedia.org/wiki/File:Frouzet_fg01.jpg

Description:

Photograph of robber fly with prey, found by searching the category "Asilidae with prey" on Wikimedia Commons. Both the robber fly and prey have a taxonomic identification (not necessarily to species).

Example 21

Taxonomy: Diptera: Asilidae: *Efferia* sp.

Record type: specimen

Life stage(s): adult

Source: iDigBio

Record URL: https://www.idigbio.org/portal/records/19dd8e8d-9540-4e5f-a360-bab5872559c4

Label image: http://collections.nmnh.si.edu/media/index.php?irn=11697090

Comments:

No label text, but the adult fly is pinned with its antlion prey.

Example 22

Taxonomy: Diptera: Asilidae: *Neoitamus angusticornis*

Record type: photograph

Life stage(s): adult

Source: Wikimedia Commons

Photograph: https://commons.wikimedia.org/wiki/File:Magarikemusihiki_08f9475c.jpg

Description:

Photograph of robber fly with prey, found by searching the category "Asilidae with prey" on Wikimedia Commons. Both the robber fly and prey have a taxonomic identification (not necessarily to species).

Example 23

Taxonomy: Diptera: Asilidae: *Orthogonis* sp.

Record type: specimen

Life stage(s): adult

Source: iDigBio

Record URL: https://www.idigbio.org/portal/records/86b0357b-5ce6-49d9-8cfd-ca29467baa78

Label image: http://collections.nmnh.si.edu/media/index.php?irn=12083105

Comments:

No label text, but a male/female pair is pinned together.

Example 24

Taxonomy: Diptera: Asilidae: *Pegesimallus* sp.

Record type: photograph

Life stage(s): adult

Source: Wikimedia Commons

Photograph: https://commons.wikimedia.org/wiki/File:Pegesimallus_sp_robberfly.jpg

Description:

Photograph of robber fly with prey, found by searching the category "Asilidae with prey" on Wikimedia Commons. Both the robber fly and prey have a taxonomic identification (not necessarily to species).

Example 25

Taxonomy: Diptera: Asilidae: *Promachus nigrialbus*

Record type: photograph

Life stage(s): adult

Source: Wikimedia Commons

Photograph: https://commons.wikimedia.org/wiki/File:Promachus_nigrialbus_female.jpg

Description:

Photograph of robber fly with prey, found by searching the category "Asilidae with prey" on Wikimedia Commons. Both the robber fly and prey have a taxonomic identification (not necessarily to species).

Example 26

Taxonomy: Diptera: Asilidae: *Tolmerus cowini*

Record type: photograph

Life stage(s): adult

Source: Wikimedia Commons

Photograph: https://commons.wikimedia.org/wiki/File:Tolmerus_20050808_073_part.jpg

Description:

Photograph of robber fly with prey, found by searching the category "Asilidae with prey" on Wikimedia Commons. Both the robber fly and prey have a taxonomic identification (not necessarily to species).

Example 27

Taxonomy: Diptera: Asilidae: *Triorla interrupta*

Record type: photograph

Life stage(s): adult

Source: Wikimedia Commons

Photograph: https://commons.wikimedia.org/wiki/File:Robber_Fly_(Triorla_interrupta)_with_Dragonfly_(Plathemis_lydia).jpg

Description:

Photograph of robber fly with prey, found by searching the category "Asilidae with prey" on Wikimedia Commons. Both the robber fly and prey are identified to species.

Example 28

Taxonomy: Diptera: Asilidae: *Triorla striola*

Record type: specimen

Life stage(s): adult

Source: iDigBio

Record URL: https://www.idigbio.org/portal/records/3a45012f-d317-47f1-9e80-9009d2386a55

Label image: http://collections.nmnh.si.edu/media/index.php?irn=11712766

Comments:

No label text, but the fly has a large ant on top of it. Not clear what the nature of the relationship was.

Example 29

Taxonomy: Diptera: Calliphoridae: *Calliphora vomitoria*

Record type: specimen

Life stage(s): adult

Source: iDigBio

Record URL: https://www.idigbio.org/portal/records/2d1e95cf-43ea-4af0-b53b-c6655eab997b

Label image: https://api.idigbio.org/v2/media/950a91df7a461ac0ffa5846800a3a1ae?size=fullsize

Relevant label text:

"rotten liver bait"

Example 30

Taxonomy: Diptera: Calliphoridae: *Calliphora vomitoria*

Record type: literature

Life stage(s): adult, larva, pupa, egg

Source: Grassberger and Frank (2004)

Relevant text:

Has species-level observations of adults, larvae, pupae and eggs from two specimens allowed to decompose over 60 days. Includes information by decomposition stage and by days since death. E.g., Calliphora vomitoria observed as adults and eggs on one specimen of Sus scrofa freshly decomposed (from table 1).

Example 31

Taxonomy: Diptera: Calliphoridae: *Chrysomya megacephala*

Record type: specimen

Life stage(s): adult

Source: iDigBio

Record URL: https://www.idigbio.org/portal/records/4d1702d5-6632-47d5-906c-c42088c35ae9

Label image: https://api.idigbio.org/v2/media/71eeadb7d0bc5fda2b7da9d93d060840?size=fullsize

Relevant label text:

"captured on dead yearling calf"

Example 32

Taxonomy: Diptera: Calliphoridae: *Chrysomya megacephala*

Record type: literature

Life stage(s): adult

Source: Raju and Ezradanam (2002)

Relevant text:

"Table 1. List of flower visitors and forage collected by them on J. [Jatropha] curcas"

Example 33

Taxonomy: Diptera: Calliphoridae: *Dyscritomyia fasciata*

Record type: specimen

Life stage(s): adult

Source: iDigBio

Record URL: https://www.idigbio.org/portal/records/ca9c7b99-ff94-47f3-a5a1-69ab360da77c

Label image: https://api.idigbio.org/v2/media/21f11b72b427964dccb677f4be7286f2?size=fullsize

Relevant label text:

"trap 207 (BioLure) | fruit fly nontarget research"

Example 34

Taxonomy: Diptera: Calliphoridae: *Phormia regina*

Record type: literature

Life stage(s): adult, larva, pupa, egg

Source: Anderson and VanLaerhoven (1996)

Relevant text:

Has species-level observations of adults, larvae, pupae and eggs from seven Sus scrofa carcases in British Colombia by decomposition stage. E.g., Phormia regina was observed as adult and immature at the Bloat (2-10d) stage of decomposition. Also has number of individuals found under carcass.

Example 35

Taxonomy: Diptera: Calliphoridae, Sarcophagidae, Muscidae, Phoridae, Piophilidae, Sepsiddae, Asilidae, Stratiomyidae

Record type: literature

Life stage(s): adult, larva, pupa, egg

Source: Sukchit et al. (2015)

Relevant text:

Decomposition stage identifications are only available at the family rank. Number of individuals at the species rank are available across three seasons (monsoon wet, winter, summer) for mixed deciduous forest and suburban area.

Example 36

Taxonomy: Diptera: Cecidomyiidae

Record type: specimen

Life stage(s): larva

Source: iDigBio

Record URL: https://www.idigbio.org/portal/records/c972f72f-1815-488c-8b16-15d9dd782835

Label image: https://invertnet.org/imagestor/vials/larges/2014/11/13/11260-0.jpg

Relevant label text:

"in oak leaf gall"

Example 37

Taxonomy: Diptera: Cecidomyiidae: *Parallelodiplosis cattleyae*

Record type: specimen

Life stage(s): unknown

Source: iDigBio

Record URL: https://www.idigbio.org/portal/records/eb7e214e-b5d9-499c-9ea0-95d5c8be2f35

Label image: https://api.idigbio.org/v2/media/1cca23beaa7bbffbf979fdd1d9734761?size=fullsize

Relevant label text:

"galls on aerial roots of orchids"

Example 38

Taxonomy: Diptera: Chironomidae: *Corynoneura scutellata*

Record type: literature

Life stage(s): larva

Source: Lencioni et al. (2012)

Relevant text:

"Seventeen chironomid species were present in > 30 springs, 42 species were present in < 10 springs, and 9 species occurred in only 1 spring. From 1 to 32 species were identified per spring. Twenty springs had > 30 species, and 15 springs had < 5 species. Widespread species had higher abundance than species with restricted distributions. The most frequent species were Tvetenia calvescens, Corynoneura scutellata, Metriocnemus eurynotus gr., and Micropsectra atrofasciata gr., which were present in >60 springs."

Example 39

Taxonomy: Diptera: Culicidae: *Anopheles gambiae*

Record type: literature

Life stage(s): adult

Source: Charlwood et al. (2002)

Relevant text:

"Swarms were seen on all of the 194 evenings and on 9 of 14 mornings that observations took place. Swarming at dusk was related to the time of sunset, which in São Tomé over the year varies between 17:20 and 17:50 (Figure 2). Males started swarming 2 min before sunset at sheltered sites (sites 1,2 and 4 in Figure 1) (start time = 0.92 time of sunset + 0.37min; R 2 =92%) and a minute or two later at the more exposed site 3 (start time = 0.88 time of sunset + 1.74 min; R 2 =75%). Males arrived at swarming sites 1 to 2m above the ground, along edges of contrast such as the grass-path edge with occasional zigzag flights over such things as tufts of grass until they ended up at the usual swarm site. Within the area described in Figure 1 up to six swarms could occur on a single night. All swarms occurred at least 2.5m, and in one site 4m, off the ground, over the darker sides of two edges of horizontal contrast, one of which was generally a minimum of 1.50m long."

"Swarming ended 15 min after dawn."

Example 40

Taxonomy: Diptera: Culicidae: *Culex* sp.

Record type: database

Life stage(s): adult

Source: Chicago Department of Public Health (2018)

Comments and relevant content:

Records the numbers of mosquitoes found in traps in Chicago between 2007 and 2018 and whether they were positive or negative for West Nile Virus. Does not include absence data, i.e. every record has at least one mosquito. Some example data records (in CSV format):

SEASON YEAR,WEEK,TEST ID,BLOCK,TRAP,TRAP_TYPE,TEST DATE,NUMBER OF MOSQUITOES,RESULT,SPECIES,LATITUDE,LONGITUDE,LOCATION

2017,29,44972,37XX E 118TH ST,T212,GRAVID,2017 Jul 20 12:07:00 AM,5,negative,CULEX TERRITANS,41.681034931,-87.533436467,(41.681034931122895°, -87.53343646731054°)

2017,28,44767,22XX W 69TH ST,T069,GRAVID,2017 Jul 14 12:07:00 AM,10,positive,CULEX RESTUANS,41.768485667,-87.679463428,(41.768485667016584°, -87.67946342813606°)

Example 41

Taxonomy: Diptera: Muscidae: *Musca domestica*

Record type: literature

Life stage(s): adult

Source: Mumcuoglu and Braverman (2010)

Relevant text:

"Most house flies (M. domestica L.) and stable flies (S. calcitrans L.) were infested by the mesostigmatid mite Macrocheles muscaedomesticae (Macrochelidae), and a smaller number were infested by nymphs of M. subbadeus Berlese and Dendrolaelaps sp. (Digamasselidae), as well as by trombiculid larvae (Trombiculidae)."

Example 42

Taxonomy: Diptera: Mycetophilidae: *Arachnocampa, Orfelia*

Record type: literature

Life stage(s): larva

Source: Viviani et al. (2002)

Relevant text:

"The bioluminescence of Orfelia is blue, and that of Arachnocampa is blue-green."

"Although the luminescence originates from the anterior and posterior lanterns, a diffuse bioluminescence is often observable throughout the body. When larvae were manipulated, we sometimes noticed that bioluminescent material was released, either as a secretion or as the result of injury."

Example 43

Taxonomy: Diptera: Sarcophagidae

Record type: specimen

Life stage(s): larva

Source: iDigBio

Record URL: https://www.idigbio.org/portal/records/f3ca95ac-e557-439c-851d-d40e857b406b

Label image: https://invertnet.org/imagestor/vials/larges/2014/11/21/11486-0.jpg

Relevant label text:

"on wounds of live rabbit"

Example 44

Taxonomy: Diptera: Sarcophagidae

Record type: specimen

Life stage(s): larva

Source: iDigBio

Record URL: https://www.idigbio.org/portal/records/f3ca95ac-e557-439c-851d-d40e857b406b

Label image: https://invertnet.org/imagestor/vials/larges/2014/11/21/11486-0.jpg

Relevant label text:

"in cow dung"

Example 45

Taxonomy: Diptera: Sarcophagidae
Record type: specimen
Life stage(s): larva
Source: iDigBio
Record URL: https://www.idigbio.org/portal/records/f3ca95ac-e557-439c-851d-d40e857b406b
Label image: https://invertnet.org/imagestor/vials/larges/2014/11/21/11486-0.jpg
Relevant label text:

"found under the bark of dead white oak tree"

Example 46

Taxonomy: Diptera: Sarcophagidae
Record type: specimen
Life stage(s): larva
Source: iDigBio
Record URL: https://www.idigbio.org/portal/records/8ee08ca9-2ac9-48a1-b361-c19eb535fc74
Label image: https://invertnet.org/imagestor/vials/larges/2014/11/21/11484-0.jpg
Relevant label text:

"from opossum"

Example 47

Taxonomy: Diptera: Sarcophagidae
Record type: specimen
Life stage(s): larva
Source: iDigBio
Record URL: https://www.idigbio.org/portal/records/8ee08ca9-2ac9-48a1-b361-c19eb535fc74
Label image: https://invertnet.org/imagestor/vials/larges/2014/11/21/11484-0.jpg
Relevant label text:

"in dead frog"

Example 48

Taxonomy: Diptera: Sarcophagidae
Record type: specimen
Life stage(s): larva
Source: iDigBio
Record URL: https://www.idigbio.org/portal/records/8ee08ca9-2ac9-48a1-b361-c19eb535fc74
Label image: https://invertnet.org/imagestor/vials/larges/2014/11/21/11484-0.jpg
Relevant label text:

"in decaying manure & vegetable material"

Example 49

Taxonomy: Diptera: Sarcophagidae
Record type: specimen
Life stage(s): larva
Source: iDigBio
Record URL: https://www.idigbio.org/portal/records/8ee08ca9-2ac9-48a1-b361-c19eb535fc74
Label image: https://invertnet.org/imagestor/vials/larges/2014/11/21/11484-0.jpg
Relevant label text:

"road kill"

Example 50

Taxonomy: Diptera: Sarcophagidae
Record type: specimen
Life stage(s): larva
Source: iDigBio
Record URL: https://www.idigbio.org/portal/records/ee40a40b-5f75-4f08-8ea9-70e7ee951b70
Label image: https://invertnet.org/imagestor/vials/larges/2014/11/21/11484-1.jpg
Relevant label text:

"ex dead cat"

Example 51

Taxonomy: Diptera: Sarcophagidae
Record type: specimen
Life stage(s): larva
Source: iDigBio
Record URL: https://www.idigbio.org/portal/records/ee40a40b-5f75-4f08-8ea9-70e7ee951b70
Label image: https://invertnet.org/imagestor/vials/larges/2014/11/21/11484-1.jpg
Relevant label text:

"ex dead skunk | (in the middle of the road?) | stinkin' to high heaven"

Example 52

Taxonomy: Diptera: Sarcophagidae
Record type: specimen
Life stage(s): larva
Source: iDigBio
Record URL: https://www.idigbio.org/portal/records/ee40a40b-5f75-4f08-8ea9-70e7ee951b70
Label image: https://invertnet.org/imagestor/vials/larges/2014/11/21/11484-1.jpg
Relevant label text:

"live rabbit"

Example 53

Taxonomy: Diptera: Sarcophagidae: *Acanthodotheca exuberans*

Record type: specimen

Life stage(s): adult

Source: CNC

Relevant label text:

"reared from Melanoplus mexicanus | newly dead and dying M. m. mexicanus | pupae 4th Aug. | flies 22-25 Aug."

Example 54

Taxonomy: Diptera: Sarcophagidae: *Agria housei*

Record type: specimen

Life stage(s): adult

Source: CNC

Relevant label text:

"reared from female C. pellucida | On Sept. 12, 1941 this fly emerged from a female C. pellucida collected Aug. 19, 1941. The female C. pellucida also produced a complete egg pod."

Example 55

Taxonomy: Diptera: Sarcophagidae: *Argorivinia spinosa*

Record type: literature

Life stage(s): adult

Source: Lopes (1988)

Relevant text:

"Flies from St. Agostine, Trinidad, were found associated with nest of Sphecidae (Hymenoptera)."

Example 56

Taxonomy: Diptera: Sarcophagidae: *Argorivinia spinosa*

Record type: literature

Life stage(s): adult

Source: Coffey (1966)

Relevant text:

Table 2 details a bunch of fly species collected and/or reared on dung. Gives numbers of flies collected and reared on dung from 7 mammal species, with separate counts for each dung type.

Example 57

Taxonomy: Diptera: Sarcophagidae: *Blaesoxipha aculeata*

Record type: specimen

Life stage(s): adult

Source: CNC

Relevant label text:

"progeny ex female collected Chatterton, Ont. | host: Melanoplus mexicanus"

Example 58

Taxonomy: Diptera: Sarcophagidae: *Blaesoxipha aculeata*
Record type: specimen
Life stage(s): adult
Source: CNC
Relevant label text:

"16 days in pupal stage | temp 74° F"

Example 59

Taxonomy: Diptera: Sarcophagidae: *Blaesoxipha aculeata*
Record type: specimen
Life stage(s): adult
Source: CNC
Relevant label text:

"lab bred | larva to adult 27 days | 74° F"

Example 60

Taxonomy: Diptera: Sarcophagidae: *Blaesoxipha hunteri*
Record type: specimen
Life stage(s): adult (with puparium)
Source: CNC
Relevant label text:

"among locust eggs"

Example 61

Taxonomy: Diptera: Sarcophagidae: *Blaesoxipha impar*
Record type: specimen
Life stage(s): adult
Source: CNC
Relevant label text:

"bred from decaying meat"

Example 62

Taxonomy: Diptera: Sarcophagidae: *Blaesoxipha impar*

Record type: literature

Life stage(s): larva

Source: Hall (1929)

Relevant text:

"The technique employed to secure these parasites was to collect the living beetles [Phyllophaga] in the field, placing them in fly-tight rearing cages at the insectary. These cages were inspected daily, emerged material and dead beetles being removed.

Either the Sarcophagids which emerged were parasitic upon the living beetles, or they were scavengers upon dead beetles. All the material placed in the rearing cages was alive at the time of entering. Therefore there was no chance of introducing scavengers at that time. Female Sarcophagids may have dropped maggots through the screenwire upon dead beetles. This is a very faint possibility, for the dead beetles were removed from the cages daily, and several hours must elapse after the beetles are dead before the beetle becomes putrified enough to become an attrahent to female Sarcophagids. This time varies with the temperature and humidity, but it hardly seems probable that a dead beetle would be blown before twenty-four hours. For these reasons, it is felt that the species are true parasites upon the species of Phyllophaga concerned.

…

The species [B. impar] has been reared from grasshoppers, and several kinds of refuse. Professor McColloch's records show that it has been reared from adult Phyllophaga lanceolata."

Example 63

Taxonomy: Diptera: Sarcophagidae: *Blaesoxipha impar*

Record type: literature

Life stage(s): larva

Source: Aldrich (1916)

Relevant text:

"one [specimen from] Greensboro, Fla., reared from the large lubber grasshopper, Dictyophorus reticulatus, by R. N. Wilson (Gainesville, No. 16587) … One lot was bred from beef refuse at Victoria, Texas, by J. D. Mitchell. Mr. E. G. Kelly reports that the Big Cabin specimen was reared from pupae of Heliophila unipuncta, which were living when placed in the cage."

Example 64

Taxonomy: Diptera: Sarcophagidae: *Blaesoxipha impar*

Record type: literature

Life stage(s): larva

Source: Graenicher (1931)

Relevant text:

"The species mentioned below have been bred from dead animals and excrements exposed in the open in my immediate neighborhood, or from material picked up at random at various points in the Miami region. … S. impar Ald. from 1 (landcrab)"

Example 65

Taxonomy: Diptera: Sarcophagidae: *Blaesoxipha impar*

Record type: literature

Life stage(s): larva

Source: Roberts (1933b)

Relevant text:

"Sarcophaga impar Ald. – Dallas, Tex. August 24, 1928, larvae taken from dead bird, placed on beef, and exposed to parasites. September 13-14, 11 S. impar (identified by Laake) emerged."

Example 66

Taxonomy: Diptera: Sarcophagidae: *Blaesoxipha impar*

Record type: literature

Life stage(s): larva

Source: Roback (1954)

Relevant text:

"has been reared from garbage by the author"

Example 67

Taxonomy: Diptera: Sarcophagidae: *Blaesoxipha impar*

Record type: literature

Life stage(s): larva

Source: Knipling (1936)

Relevant text:

"Reared on decaying meats."

Example 68

Taxonomy: Diptera: Sarcophagidae: *Blaesoxipha impar*

Record type: literature

Life stage(s): larva

Source: Downes (1965)

Relevant text:

"The members of this subgenus are not so strictly parasitic as other members of the genus. Rearing records include adult beetles, grasshoppers, carrion, and old wounds in mammals."

Example 69

Taxonomy: Diptera: Sarcophagidae: *Blaesoxipha plinthopyga*

Record type: specimen

Life stage(s): adult

Source: CNC

Relevant label text:

"causing myiasis in cattle"

Example 70

Taxonomy: Diptera: Sarcophagidae: *Blaesoxipha plinthopyga*

Record type: literature

Life stage(s): larva

Source: Aldrich (1916)

Relevant text:

"A large series of specimens from Texas collected and reared from carrion by F. C. Bishopp and associates. … Mr. Bishopp says in regard to the habits (in a letter), "A number of lots were bred from carcasses of animals and exposed beef.""

Example 71

Taxonomy: Diptera: Sarcophagidae: *Blaesoxipha plinthopyga*

Record type: literature

Life stage(s): larva

Source: Denno and Cothran (1976)

Relevant text:

"In order to sample the population size of each fly species which successfully exploited a carcass, a device was designed to collect larvae as they emigrated from the carrion to pupate. The sampling unit (Fig. 1) consisted of a shallow wooden tray, 70 cm square and 20 cm deep with a flange projecting beyond the upper rim. Five cm of fine white aquarium sand was spread over the bottom of the tray. A removable wooden platform covered with 1 cm of sand, on which the carcass rested, was then nestled into the sand that covered the bottom of the wooden tray. Domestic white rabbit carcasses (~1.0 kg) were used for all experiments. To prevent vertebrate scavengers from disturbing the carcass, a large mesh (2.50x0.75 cm) galvanized wire cage ("carcass protector") was fitted within the flange that surrounded the top of the tray and held in place by hook-springs. The arrangement allowed adult flies to freely visit and leave the carcass for feeding and oviposition.

After larval development is complete, maggots emigrate from the carcass within the field unit, drop off the edge of the platform and tunnel into the sand beneath. The "carcass-protector," the "excluder," if present, and the wooden platform holding the carcass were respectively removed, and the maggots sifted from the remaining sand. The field box was then reassembled and the larvae were returned to the laboratory. This sifting procedure was repeated daily until most maggots left the carcass. After all food resources were depleted, there were usually a few undernourished larvae remaining under the hide. These were removed by hand, returned to the laboratory, and sampling was considered complete.

In order to insure accurate fly identification, larvae collected from each carcass were brought to the laboratory and isolated in pupation cages that were stored at 22±1°C. The cages consisted of 2 chambers, each constructed from a gallon ice cream carton. Maggots were placed in the bottom pupation chamber and covered with either damp aquarium sand or vermiculite. An inverted plastic screen funnel separated the lower and upper chambers and prevented emerging flies from returning to the pupation chamber. Organdy gauze covered the top chamber to provide ventilation. As adult flies emerged, they crawled from the pupation chamber through the screen funnel into the upper chamber which could be removed. The enclosed flies were anesthetized with carbon dioxide and subsequently killed with chloroform.

…

Exploiting carrion contemporaneously with the calliphoids [sic] were 3 uncommon sarcophagids, Blaesoxipha plinthopyga (Wiedemann), Sarcophaga cooleyi Parker and Sarcophaga argyrostoma Robineau-Desvoidy, which together comprised ~1.0% of all flies reared from a carcass. "

Example 72

Taxonomy: Diptera: Sarcophagidae: *Blaesoxipha plinthopyga*

Record type: literature

Life stage(s): larva

Source: Laake (1936)

Relevant text:

"Table 1 gives the number and species of flies reared from each of 114 cases of myiasis occurring in domestic animals on one ranch in southwestern Texas during the year 1934. These animals became naturally infested on the range and were brought into the corral for treatment. Most of the cases were found before the infestation became extensive and before the maggots first infesting the wound had matured. None of the cases recorded was a reinfestation; that is, the wound from which the maggots were taken had not been previously treated for an infestation." Table 1 lists 12 cases of myiasis involving B. plinthopyga.

Example 73

Taxonomy: Diptera: Sarcophagidae: *Blaesoxipha plinthopyga*

Record type: literature

Life stage(s): larva

Source: James (1947)

Relevant text:

"The larvae [of B. plinthopyga] differ in their feeding habits and are commonly found on carcasses or as parasites in the bodies of insects. However, they frequently attack old and festered sores in man and animals, or invade diseased body openings. According to Patton this is a notorious myiasis-producing species in British Guiana."

Example 74

Taxonomy: Diptera: Sarcophagidae: *Blaesoxipha plinthopyga*

Record type: literature

Life stage(s): larva

Source: Roberts (1933a)

Relevant text:

"These instances occurred in 1930, and since then two additional cases of infestations of gunshot wounds have been observed. These are here described in detail, the Sarcophaga being determined by David G. Hall.

As soon as the rabbits in the two cases were killed collections of larvae from the wounds, together with the flesh surrounding the affected area, were immediately removed from the animal and placed in quart Mason jars containing sifted sand. These jars were capped with lids of 60-mesh wire cloth and placed in fly-proof cages in a fly-proof insectary. No subsequent fly infestation was therefore possible.

Case 1. On July 24, 1931, an adult jack rabbit weighing 6 1/2 pounds was killed in dense mesquite brush. An infested wound 30 by 50 mm. was found on the left rump at the base of the tail, extending through the fleshy portion to the inside of the leg where it again opened. It was apparently caused by a small-caliber rifle. Some of the fly larvae present were mature, and it appeared that others had migrated. This suggested an old wound. A seropurulent discharge was occurring, and the wound had an extremely foul odor. The larvae had worked entirely through the leg, and additional fly eggs had been deposited recently on the side of the wound. It was apparent that the rabbit would have died. The following adult specimens were reared from the larvae which remained in the wound: August 5 to 7, 35 Cochliomyia macellaria Fab.; August 15, 1 Sarcophaga plinthopyga Wied.

Case 2. On May 20, 1932, an adult jack rabbit was killed in a dense growth of mesquite and cactus. An injury in the head, another on a rear leg, and roughened spots on other parts of the body indicated that the rabbit had been previously wounded with a shotgun. The rabbit was greatly emaciated. The wound, 45 mm. in diameter, in the lower right side of the face, penetrated through skin and flesh into the interior of the mouth, extending up to about 25 mm. below the eye, and included the right nostril. There was no discharge or pus although the wound appeared old. There were many small maggots and some larger ones; a fresh lot of eggs had been deposited on the margin of the wound. The rabbit apparently would have died. The emergence from the larvae collected was as follows: May 31 to June 1, 16 C. macellaria; June 6 to 15, 583 S. plinthopyga; and June 13-14, 2 Brachymeria fonscolombei (Dufour)."

Example 75

Taxonomy: Diptera: Sarcophagidae: *Blaesoxipha savoryi*

Record type: specimen

Life stage(s): adult

Source: CNC

Relevant label text:

"em. (incubator) | host: adult Polyphylla perversa"

Example 76

Taxonomy: Diptera: Sarcophagidae: *Boettcheria cimbicis*

Record type: specimen

Life stage(s): adult

Source: CNC

Relevant label text:

"stems of wild parsnip | larvae in dead Depressaria pupae & exuviae"

Example 77

Taxonomy: Diptera: Sarcophagidae: *Boettcheria latisterna*
Record type: specimen
Life stage(s): adult
Source: CNC
Relevant label text:

"June beetle ad. parasite"

Example 78

Taxonomy: Diptera: Sarcophagidae: *Brachicoma setosa*
Record type: specimen
Life stage(s): adult
Source: CNC
Relevant label text:

"from P. perplexis bee colony"

Example 79

Taxonomy: Diptera: Sarcophagidae: *Emblemasoma auditrix*
Record type: specimen
Life stage(s): adult
Source: CNC
Relevant label text:

"larv. ex. jack pine by beating"

Example 80

Taxonomy: Diptera: Sarcophagidae: *Emblemasoma erro*

Record type: literature

Life stage(s): adult

Source: Stucky (2015)

Relevant text:

"I was able to determine the moment of larviposition for 15 of these attacks. Of these, five (33.3%) occurred while the cicada was in flight; the remaining ten attacks (66.7%) occurred while the cicada was either walking, flapping its wings, or both."

"Of 15 attacks for which the exact location of larviposition was determined (out of 17 total successful attacks), 1 (6.7%) was on the base of a fore leg, 2 (13.3%) were on the abdomen, and 12 (80%) were either directly on the wings (usually near the base) or on the pterothorax or the first two abdominal segments next to the base of the wings."

"The number of larvae deposited by a single female fly on a host cicada during the infection behavior trials (i.e., the clutch size) varied from a minimum of one to a maximum of six, but more than 80% of the time, flies (14 of 17) deposited three or fewer larvae. The mean clutch size was 2.53 (95% bootstrap-t CI: 1.85 to 3.45 larvae/host, s = 1.50, n = 17 hosts) and the median was 3."

"From the moment of larviposition until they completely exited the host's body, E. erro larvae spent, on average, 88.0 h residing inside their host (95% CI: 81.19 to 94.76 h, s = 17.1, n = 27 larvae from 13 host cicadas and 10 female flies, range = 61.3 to 116.0)."

"The exact location of egress was observed for 83 larvae from 28 T. dorsatus hosts, and of these, 64 (77.1%) exited from behind one of the cicada's opercula. Of the remaining larvae, 16 (19.3%) exited next to the pygofer or terminal abdominal segments at the apex of the abdomen, and 3 (3.6%) burrowed through the membrane between the head and prothorax."

"Adult flies eclosed 18.4 days, on average, after leaving their host (95% CI: 18.02 to 18.69 days, s = 0.91, n = 31 flies from 15 host cicadas, range = 16 to 20 days). The lifespan of adult flies in the field is unknown. Adult flies maintained in the laboratory survived as long as 92 days."

"These flies carried as few as 3 and as many as 174 larvae in their incubatory pouches, with a mean of 60.7 larvae per fly (s = 57.5). The observed distribution of larvae counts was strikingly bimodal: Three flies had more than 150 larvae, while all of the rest had fewer than 80. The larvae of the four flies with the largest larvae counts were noticeably smaller than those from the remaining flies and generally had less well-developed bristles. Remnants of eggshell were still visible in the incubatory pouches of three of these flies, suggesting that the larvae had recently hatched."

"Across all four sampling years (2011 to 2014) and all six primary study sites, the overall observed parasitism rate for T. dorsatus males was 26.3% (95% CI: 21.4 to 31.9%, n = 266 cicadas). The surveys in 2014 also included a sample of 28 female T. dorsatus from the central KS field sites (in Harvey, McPherson, and Reno counties), and of these, one female cicada was infected with E. erro larvae (3.7%; 95% CI: 0.7% to 18.3%)."

"The mean parasitoid load of all field-collected infected cicadas was 4.97 larvae/host (95% bootstrap-t CI: 4.23 to 5.92 larvae/host, s = 3.95, n = 91 hosts, range = 1–19 larvae/host) and the median was 4, reflecting the strong right skew of the distribution."

Example 81

Taxonomy: Diptera: Sarcophagidae: *Emblemasoma erro*

Record type: literature

Life stage(s): adult

Source: Stucky (2016)

Relevant text:

"Two matings involved male and female files that were observed copulating inside one of the live traps after both had been captured. The durations of these matings were not precisely timed, but in the second case, the pair remained together for at least 25 min. In the third observation of mating in the field, two flies that landed near the loudspeaker began copulating, then flew off joined together. Attempts were also made to observe mating behavior in captivity in the lab, but these efforts were mostly unsuccessful. However, putative male mating behavior in captivity was similar to that observed at the sound broadcasts in the field, with males pursuing potential mates either by walking or by flight. Only one successful mating was observed in captivity, between a pair of flies that had been reared from a parasitized N. dorsatus (Fig. 2). These flies remained in copula for at least 90 min."

"All results of this study were consistent with the hypothesis that Emblemasoma erro uses the acoustic sexual signals of its hosts as a means for locating potential mates. In the field, both male flies and nongravid female flies performed positive phonotaxis to acoustic stimuli mimicking the calls of their host cicadas. Unmated female flies with no previous exposure to male flies or host signals were also phonotactically responsive. Once male flies arrived at a sound source, they pursued and tried to mate with other flies that were also attracted to the acoustic stimulus."

Example 82

Taxonomy: Diptera: Sarcophagidae: *Fletcherimyia papei*

Record type: literature

Life stage(s): larva

Source: Dahlem and Naczi (2006)

Relevant text:

"F. papei seems to be associated with the pitcher plant species S. rubra (including the subspecies wherryi)"

"Type. Holotype male [FSCA]. Labels on holotype: (1) ALABAMA: Washington Co.; 4.2 miles. S of Chatom, Route 17; 3-VIII-1994; R.F.C. Naczi; ex: Sarracenia rubra subsp. wherryi;"

Example 83

Taxonomy: Diptera: Sarcophagidae: *Mantidophaga blandita*

Record type: specimen

Life stage(s): adult

Source: CNC

Relevant label text:

"Parasita do Mantodea no. 34 | saida larva: 25/Fev. | Pupa: 26/Fev. | Imago: 13/Mar/1959"; translation: "parasite of Mantodea no. 34 | larval exit: 25/Feb. | Pupa: 26/Feb. | Imago: 13/Mar./1959"

Example 84

Taxonomy: Diptera: Sarcophagidae: *Metoposarcophaga importuna*
Record type: specimen
Life stage(s): adult
Source: MCZ
Relevant label text:

"parasitoid of Malaclemys t. terrapin (egg) | ex pupa collected from nest #2 | emerged in lab: 18 IV 1980"

Example 85

Taxonomy: Diptera: Sarcophagidae: *Miltogramma erythrura*
Record type: specimen
Life stage(s): adult
Source: CNC
Relevant label text:

"emerged from Anthophora cell"

Example 86

Taxonomy: Diptera: Sarcophagidae: *Ravinia derelicta*
Record type: specimen
Life stage(s): adult
Source: iDigBio
Record URL: https://www.idigbio.org/portal/records/a86dea6b-43b5-4362-b2c2-a6c3e1ed285d
Label image: https://api.idigbio.org/v2/media/fee3c9cd14cfd07482bc4f3cf310352c?size=fullsize
Relevant label text:

"on flower of Ximenia americana | scrubby flatwoods"

Example 87

Taxonomy: Diptera: Sarcophagidae: *Sarcofahrtiopsis paterna*
Record type: specimen
Life stage(s): adult
Source: iDigBio
Record URL: https://www.idigbio.org/portal/records/97c5c4a9-80d0-4815-a68a-02ec20b12806
Label image: https://api.idigbio.org/v2/media/17be9b33102a6a1289bb8e5ad08c587b?size=fullsize
Relevant label text:

"on staminate flower of Salix caroliniana"

Example 88

Taxonomy: Diptera: Sarcophagidae: *Sarcophaga africa*
Record type: specimen
Life stage(s): adult
Source: MCZ
Relevant label text:

"bred from human excrement"

Example 89

Taxonomy: Diptera: Sarcophagidae: *Sarcophaga citellivora*

Record type: specimen

Life stage(s): adult

Source: CNC

Relevant label text:

"reared from Spermophilus richardsonii (moribund, host no. 1760) | 28.vi.2002, emerged 16-19.viii.2002"

Example 90

Taxonomy: Diptera: Sarcophagidae: *Sarcophaga cooleyi*

Record type: specimen

Life stage(s): adult

Source: CNC

Relevant label text:

"ex. scalp wound in child"

Example 91

Taxonomy: Diptera: Sarcophagidae: *Sarcophaga cooleyi*

Record type: specimen

Life stage(s): adult

Source: CNC

Relevant label text:

"reared | emerged 15-III-55 | from ear of Indian boy at Camsell Hosp. Edmonton. Oct. 7/54. Pupated enroute | emerged Kamloops 13-III-55"

Example 92

Taxonomy: Diptera: Sarcophagidae: *Sarcophaga dux sarracenioides*

Record type: specimen

Life stage(s): larva

Source: iDigBio

Record URL: https://www.idigbio.org/portal/records/f3ca95ac-e557-439c-851d-d40e857b406b

Label image: https://invertnet.org/imagestor/vials/larges/2014/11/21/11486-0.jpg

Relevant label text:

"in pitcher plant"

Example 93

Taxonomy: Diptera: Sarcophagidae: *Sarcophaga falciformis*

Record type: literature

Life stage(s): adult

Source: Middlekauff (1959)

Relevant text:

"Ten wild female flies were dissected and the number of larvae per female counted. They varied from a low of 7 to a high of 47, with an average of 22.1 per female."

"In the sixth specimen opened a living first-instar was discovered deeply embedded in the muscles of the hind femur. That this is the normal habit for Sarcophaga falciformis was subsequently proved by dissecting over 70 specimens of Melanoplus devastator and Oedaleonotus enigma which had been attacked. These dissections also showed that only 24 percent of the attacks resulted in the deposition of a maggot."

Example 94

Taxonomy: Diptera: Sarcophagidae: *Sarcophaga libera*

Record type: specimen

Life stage(s): adult

Source: CNC

Relevant label text:

"p. 4-5 Aug. 88 | em. 24 Aug. 88 | ex Dolichovespula arenaria nest"

Example 95

Taxonomy: Diptera: Sarcophagidae: *Sphecapatodes hilarella*

Record type: specimen

Life stage(s): adult

Source: CNC

Relevant label text:

"reared from larvae in burrow of Cnemidophorus sexlineatus (lizard)"

Example 96

Taxonomy: Diptera: Syrphidae: *Microdon albicomatus*

Record type: literature

Life stage(s): larva

Source: Howard et al. (1990)

Relevant text:

"Second and third instars of Microdon albicomatus Novak and workers and brood of Myrmica incompleta Provancher were collected from a fallen log..."

Example 97

Taxonomy: Diptera: Syrphidae: *Microdon bruchi*
Record type: specimen
Life stage(s): adult
Source: iDigBio
Record URL: https://www.idigbio.org/portal/records/17562141-3f1b-47eb-a9ff-c3c525810755
Label image: http://collections.nmnh.si.edu/media/index.php?irn=12120288
Relevant label text:

"laying eggs in trail of ants"

Example 98

Taxonomy: Diptera: Syrphidae: *Microdon cothurnatus*
Record type: literature
Life stage(s): larva
Source: Akre et al. (1973)
Relevant text:

"One hundred twenty-one nests of Formica obscuripes were searched for Microdon larvae. Ten contained larvae, while 9 more had empty pupal cases from previous years."

"A total of 555 larvae was collected. Five nests contributed most of larvae with 241, 86, 64, 60, and 39. Empty pupal cases in these same nests ranged from 0-141. Several nests contained about 20 larvae, and the remainder had 1-3 larvae and several empty pupal cases."

"From December to March, Microdon larvae were found 30-60 cm deep within the ant nest, usually at the interface between the thatching of the nest and the soil or immediately beneath this interface. Larvae were usually found singly on small rocks or bits of wood at this level. Ants were present at this same level, clustered into compact masses 5-7 cm diameter. By April or early May the larvae moved near the nest surface and all had pupated by mid May. Empty pupal cases were usually near the nest surface although several were deep within the nest."

"Workers usually showed no reaction to the larvae, crawling over and about them in their normal travels (Fig. 5). However, if the ants were excited by shaking the container, they sometimes tried to bite the larvae but with no visible effect. The one exception was a larva which was partly overturned enabling workers to attack its soft underside. This was the only larva killed by ants in over 50 hours of observations. Larvae seemed attracted to the ants and slowly followed the main body of the ants whenever they shifted position within the container. Once with the ants, the larvae were quite inactive and move infrequently (Figs. 5, 6)."

Example 99

Taxonomy: Diptera: Tachinidae
Record type: specimen
Life stage(s): larva
Source: iDigBio
Record URL: https://www.idigbio.org/portal/records/e876a63a-fbdd-4afd-bd99-7a5ec9ee641d
Label image: https://invertnet.org/imagestor/vials/larges/2014/11/21/11593-1.jpg
Relevant label text:

"in notodontid larva"

Example 100

Taxonomy: Diptera: Tachinidae

Record type: specimen

Life stage(s): larva

Source: iDigBio

Record URL: https://www.idigbio.org/portal/records/e876a63a-fbdd-4afd-bd99-7a5ec9ee641d

Label image: https://invertnet.org/imagestor/vials/larges/2014/11/21/11593-1.jpg

Relevant label text:

"from Notodontidae larvae"

Example 101

Taxonomy: Diptera: Tachinidae

Record type: specimen

Life stage(s): adult

Source: iDigBio

Record URL: https://www.idigbio.org/portal/records/e876a63a-fbdd-4afd-bd99-7a5ec9ee641d

Label image: https://invertnet.org/imagestor/vials/larges/2014/11/21/11593-1.jpg

Comments and relevant label text:

"larva VIII-16-73 pupated VIII-18-73 | parasites emerged VIII-30,31-73"; note that host specimen is also present.

Example 102

Taxonomy: Diptera: Tachinidae: *Leschenaultia exul*

Record type: specimen

Life stage(s): unknown

Source: iDigBio

Record URL: https://www.idigbio.org/portal/records/62e2002c-8c81-4771-8c28-75e1b42b3965

Relevant label text:

"Voucher Specimen; Malacosoma disstria larva collected July 5, 2009. Larval parasitoid emerged July 26, 2009 from 5th instar host larva."

Hemiptera

Example 103

Taxonomy: Hemiptera: Aradidae
Record type: specimen
Life stage(s): adult
Source: iDigBio
Record URL: https://www.idigbio.org/portal/records/62400966-a82a-47b3-b7d7-c784cb879550
Relevant text and iDigBio data field(s):

"secondary growth on white sand" (habitat)

Example 104

Taxonomy: Hemiptera: Aradidae: *Aneurus inconstans*
Record type: specimen
Life stage(s): adult
Source: iDigBio
Record URL: https://www.idigbio.org/portal/records/70c45c7b-b43e-483c-80d3-8bcb653542e5
Label image: https://api.idigbio.org/v2/media/9b426adb88600af94017a408b452c513?size=fullsize
Relevant label text:

"Under Bark on Acer saccharum"

Example 105

Taxonomy: Hemiptera: Cicadidae
Record type: specimen
Life stage(s): exuvia
Source: iDigBio
Record URL: https://www.idigbio.org/portal/records/9ec29c89-3606-4573-bd80-8704dbd5531e
Comments:

No relevant label text.

Example 106

Taxonomy: Hemiptera: Cicadidae
Record type: specimen
Life stage(s): nymph
Source: iDigBio
Record URL: https://www.idigbio.org/portal/records/9612bc2a-1943-4403-9324-964d15081cec
Label image: https://invertnet.org/imagestor/vials/larges/2015/01/07/12313-0.jpg
Relevant label text:

"on lawn under elm tree"

Example 107

Taxonomy: Hemiptera: Cicadidae: *Diceroprocta apache*

Record type: specimen

Life stage(s): nymph

Source: iDigBio

Record URL: https://www.idigbio.org/portal/records/9612bc2a-1943-4403-9324-964d15081cec

Label image: https://invertnet.org/imagestor/vials/larges/2015/01/07/12313-0.jpg

Relevant label text:

"From Asparagus"

Example 108

Taxonomy: Hemiptera: Cicadidae: *Hadoa duryi*

Record type: specimen

Life stage(s): adult

Source: iDigBio

Record URL: https://www.idigbio.org/portal/records/f22210fc-8a4a-403f-9893-b98cbc37cde9

Label image: http://symbiota4.acis.ufl.edu/imglib/scan/TTU_TTU-Z/201507/TTU-Z_245013_labels_1438273809.jpg

Relevant label text:

"Pinus edulis Engelm. | Pinyon pine"

Example 109

Taxonomy: Hemiptera: Cicadidae: *Hadoa neomexicensis*

Record type: literature

Life stage(s): adult

Source: Stucky (2013)

Relevant text:

"Both Tibicen neomexicensis and Tibicen chiricahua are associated with pinyon-juniper woodlands, and neither species seems to occur in habitats where both pinyon pines (Pinus edulis, primarily) and junipers (Juniperus sp.) are absent."

"Specimen label data and field observations indicate that adults of Tibicen neomexicensis and Tibicen chiricahua emerge in early summer and are mostly gone by the end of July."

"The daily activity patterns of these two cicada species [H. neomexicensis and H. chiricahua] are also similar. Once the sun warms them sufficiently, males of both species will sing throughout much of the day, with peak calling activity occurring from about mid-day through early afternoon. Calling activity greatly diminishes during the late afternoon and evening."

"...numerous ovipositing females of Tibicen neomexicensis were observed at the type locality, most of which were placing their eggs in the dead, dried stems of grasses and forbs, often quite near to the ground."

"Both Tibicen chiricahua and Tibicen neomexicensis are commonly found with Tibicen duryi Davis, another species that is specialized on pinyon-juniper habitats."

Example 110

Taxonomy: Hemiptera: Cicadidae: *Lyristes cristobalensis*
Record type: specimen
Life stage(s): adult
Source: iDigBio
Record URL: https://www.idigbio.org/portal/records/dc318ce4-07c1-419c-90c5-36359218b0b7
Label image: http://mediaphoto.mnhn.fr/media/1441012849692EFzBakUPgKgxYqae
Relevant label text:

"Black light, coconut plantation"

Example 111

Taxonomy: Hemiptera: Cicadidae: *Magicicada* sp.
Record type: specimen
Life stage(s): nymph
Source: iDigBio
Record URL: https://www.idigbio.org/portal/records/9612bc2a-1943-4403-9324-964d15081cec
Label image: https://invertnet.org/imagestor/vials/larges/2015/01/07/12313-0.jpg
Relevant label text:

"emerged from eggs laid in twigs"

Example 112

Taxonomy: Hemiptera: Cimicidae: *Cimex serratus*
Record type: specimen
Life stage(s): adult
Source: iDigBio
Record URL: https://www.idigbio.org/portal/records/82331f41-3385-4615-98ad-72bd37fc7e32
Relevant text and iDigBio data field(s):

"ex. Pipistrellus abramus" (occurrenceRemarks)

Example 113

Taxonomy: Hemiptera: Cimicidae: *Hesperocimex coloradensis*
Record type: specimen
Source: iDigBio
Record URL: https://www.idigbio.org/portal/records/cbc1e64f-f59b-4244-b358-3d5b102dd3a0
Relevant text and iDigBio data field(s):

"abandoned woodpecker nest" (habitat)

Example 114

Taxonomy: Hemiptera: Coreidae: *Amblypelta lutescens*
Record type: specimen
Life stage(s): adult
Source: iDigBio
Record URL: https://www.idigbio.org/portal/records/0900c73c-a436-49e5-8c4b-787ad9734c92
Label image: http://www2.nau.edu/sbugs-p/UHIM/UHIM2014.04/UHIM2014.04209_lg.jpg
Relevant label text:

"Reared in Lab on Beans"

Example 115

Taxonomy: Hemiptera: Miridae
Record type: specimen
Life stage(s): adult
Source: iDigBio
Record URL: https://www.idigbio.org/portal/records/20c7dd68-6945-4eab-bfcf-eed50ee72541
Label image: http://www2.nau.edu/sbugs-p/UHIM/UHIM2014.11/UHIM2014.11865_lg.jpg
Relevant label text:

"ex dead Pritchardia frond"

Example 116

Taxonomy: Hemiptera: Miridae: *Ranzovius clavicornis*
Record type: specimen
Life stage(s): adult
Source: iDigBio
Record URL: https://www.idigbio.org/portal/records/4d5a5727-ebd9-4026-88a5-6772165ab486
Relevant label text:

"Taken on Virginia pine, in Theridiid spider web"

Example 117

Taxonomy: Hemiptera: Miridae: *Tuxedo susansolomonae*
Record type: specimen
Life stage(s): adult
Source: iDigBio
Record URL: https://www.idigbio.org/portal/records/2c7daa21-b158-40fd-9431-c156c5c9c30e
Relevant label text:

"associated with:Quercus john-tuckeri"

Example 118

Taxonomy: Hemiptera: Reduviidae: *Acanthaspis carpenteri*
Record type: specimen
Life stage(s): adult
Source: iDigBio
Record URL: https://www.idigbio.org/portal/records/6fccd3b5-6491-40c4-bb87-05ba52acac00
Label image: https://research.amnh.org/pbi/specimen/specimen/image_folder0000020/UCR_ENT%2000048469%20label.jpg
Relevant label text:

"under bark"

Example 119

Taxonomy: Hemiptera: Reduviidae: *Acanthaspis obscura*
Record type: specimen
Source: iDigBio
Record URL: https://www.idigbio.org/portal/records/9b8b13b3-8dae-4388-bf72-5d7248236781
Relevant text and iDigBio data field(s):

"Light Trap" (samplingProtocol)

Example 120

Taxonomy: Hemiptera: Reduviidae: *Acanthaspis* sp.
Record type: specimen
Source: iDigBio
Record URL: https://www.idigbio.org/portal/records/2db14e65-7923-4f8d-b0d8-6f62b13258cb
Relevant text and iDigBio data field(s):
"Under stone" (occurrenceRemarks)

Example 121

Taxonomy: Hemiptera: Reduviidae: *Apiomerus spissipes*
Record type: specimen
Source: iDigBio
Record URL: https://www.idigbio.org/portal/records/8c173112-2b8b-448a-9b3a-5088b9fcc29b
Relevant text and iDigBio data field(s):
"specimen with bee prey" (occurrenceRemarks), "Aralia spinosa" (associatedTaxa)

Example 122

Taxonomy: Hemiptera: Reduviidae: *Dicrotelus prolixus*
Record type: specimen
Life stage(s): adult
Source: iDigBio
Record URL: https://www.idigbio.org/portal/records/a36edf16-259e-4ed0-9e90-11183723d3a9
Relevant text and iDigBio data field(s):
"Host: tussocks" (occurrenceRemarks)

Example 123

Taxonomy: Hemiptera: Reduviidae: *Havinthus rufovarius*
Record type: specimen
Life stage(s): adult
Source: iDigBio
Record URL: https://www.idigbio.org/portal/records/9bf21514-6842-4761-ac0b-798d54b4e515
Relevant text and iDigBio data field(s):
"Host: under bark Eucalyptus striaticalyx" (occurrenceRemarks)

Example 124

Taxonomy: Hemiptera: Reduviidae: *Paratriatoma hirsuta*
Record type: specimen
Life stage(s): adult
Source: iDigBio
Record URL: https://www.idigbio.org/portal/records/9a2b4631-1f0b-4fec-8c55-7b00accccc3e
Relevant text and iDigBio data field(s):
"Attacking man" (occurrenceRemarks)

Example 125

Taxonomy: Hemiptera: Veliidae: *Rhagovelia boliviana*

Record type: specimen

Source: iDigBio

Record URL: https://www.idigbio.org/portal/records/34735534-8251-438a-9055-486cac053000

Relevant text and iDigBio data field(s):

"rocky stream in cloud forest " (occurrenceRemarks)

Hymenoptera

Example 126

Taxonomy: Hymenoptera: Agaonidae

Record type: specimen

Life stage(s): adult

Source: iDigBio

Record URL: https://www.idigbio.org/portal/records/532f3614-accb-4fe3-99b8-1a23042a7cdc

Label image: https://api.idigbio.org/v2/media/9588115599ad650812d2ad1792377ea8?size=fullsize

Relevant label text:

"reared ex fruits of Ficus sp."

Example 127

Taxonomy: Hymenoptera: Agaonidae: *Crossogaster michaloudi*

Record type: specimen

Life stage(s): adult

Source: iDigBio

Record URL: https://www.idigbio.org/portal/records/45d248a7-7349-423c-a8c8-844e2af36a5d

Label image: http://www.nhm.ac.uk/services/media-store/asset/a3db4a96ccedf149ae811d5d6ef71832d9f177da/contents/preview

Relevant label text:

"ex Ficus prob. artocarpoides"

Example 128

Taxonomy: Hymenoptera: Agaonidae: *Odontofroggatia galili*

Record type: specimen

Life stage(s): adult

Source: iDigBio

Record URL: https://www.idigbio.org/portal/records/79238cde-bb4c-4fd9-aad9-c13d39c6ca6d

Label image: https://api.idigbio.org/v2/media/205549d007305ef661963420961f744e?size=fullsize

Relevant label text:

"ex fruit of Ficus microcarpa"

Example 129

Taxonomy: Hymenoptera: Apidae: *Anthophorula morgani*

Record type: specimen

Life stage(s): adult

Source: iDigBio

Record URL: https://www.idigbio.org/portal/records/1ebd2fca-b085-4281-b5db-62c22f5d6a85

Label image: http://collections.nmnh.si.edu/media/index.php?irn=9117658

Relevant label text:

"on Helianthus"

Example 130

Taxonomy: Hymenoptera: Apidae: *Bombus sonorensis*

Record type: specimen

Life stage(s): adult

Source: iDigBio

Record URL: https://www.idigbio.org/portal/records/4a20faf2-a2a9-4559-a728-e57f5f186410

Label image: http://hasbrouck.asu.edu/imglib/seinet/DES/DES00036/DES00036186_lg.jpg

Comments and relevant label text:

This is an herbarium specimen of the plant Amoreuxia palmatifida. The label states, "Limestone hill, with Croton, Dasylirion wheeleri, Agave schottii, A. palmeri, Ferocactus wislizeni, Haplophyton crooksii, Talinum aurantiacum, Mimosa dysocarpa, Allionia incarnata, Bahia dissecta, Zinnia acerosa, Prosopis velutina, Cercidium floridum, Cnidoscolus angustidens, Eysenhardtia orthocarpa, Aloysia wrightii, Baccharis sarothroides, Opuntia engelmannii, , O. violacea (var. macrocentra?), Fouquieria splendens", "5 plants in immediate area, flowers with petals deep orange with purple inner bases and anthers purple, visited by Bombus (sonorensis) and many small halictids(?)."

Example 131

Taxonomy: Hymenoptera: Argidae: *Arge humeralis*

Record type: literature

Life stage(s): larva

Source: Benda et al. (2012)

Relevant text:

"In May 2009, an infestation of Arge humeralis was discovered on Metopium toxiferum [poisonwood] in the Ludlam Pineland Preserve (Miami-Dade County, Florida) by D. Powell and J. Possley. The preserve is a 4 ha remnant of unaltered pine rockland forest comprised of Pinus elliottii Engelm. var. densa (Pinales: Pinaceae), some hardwoods, and a shrub layer of saw palmetto, Serenoa repens (Bartram) J. K. Small (Arecales: Arecaceae). Due to prescribed burns, Toxicodendron radicans [poison ivy] is present at very low densities in the preserve. Metopium toxiferum is very common, but the sawfly infestation was concentrated in the western 10% of the preserve.

A cohort of 18 larvae from this infestation was sent to University of Florida, Gainesville on 22 Jun 2009 and reared in the laboratory on potted M. toxiferum in clear acrylic cylinders (20 cm diam × 60 cm high). The 8 males and 5 females that survived to adulthood served as the founder population of a laboratory colony. Females laid a total of 203 eggs, 79.8% of which hatched, beginning on 24 Jul 2009. As before, these larvae were placed on potted M. toxiferum. Plants and insects were maintained under a 15:9 h L:D photoperiod at 24.8 ± 1.0 °C and 62.4 ± 5.4% RH. Larvae that had pupated in the soil at the base of the plant were collected and individually placed into 29.6 ml plastic cups with 15 ml of moistened vermiculite until adult emergence.

Upon emergence, adults were provided potted T. radicans or M. toxiferum in clear acrylic cylinders (20 cm diam × 60 cm high) for oviposition. Insects were provided Gatorade® (0.46 mg Na, 0.13 mg P, 0.04 g sucrose, and 0.02 g fructose per mL water) (Perrone et al. 2005) in a 15 ml vial with a cotton wick. When eggs were about to eclose, the leaves containing eggs were removed and kept in Petri dishes (1.5 cm diam × 9 cm high) on moistened filter paper."

"Females readily oviposited on both T. radicans and M. toxiferum, and larval development was complete in 17.9 ± 0.6 d (N = 26) and 17.8 ± 0.6 d (N = 22), respectively (Fig. 1, F1, 46 = 0.19985, P = 0.6580). Most mortality occurred during pupation."

Example 132

Taxonomy: Hymenoptera: Bethylidae: *Goniozus jacintae.*

Record type: literature

Life stage(s): egg, larva, adult

Source: Danthanarayana (1980)

Relevant text:

"At 25 C the mean life span of the female was 15.8 days (range 6-35 days). The number of Goniozus eggs or larvae on each Epiphyas postvittana host in field samples varied from one to seven with a mean of 1.83, as follows:

Number of parasites per host (Frequency): 1 (53), 2 (16), 3 (9), 4 (7), 5 (2), 6 (1), 7 (1)"

Example 133

Taxonomy: Hymenoptera: Bethylidae: *Goniozus natalensis*

Record type: literature

Life stage(s): egg, larva, pupa, adult

Source: Conling et al. (1988)

Relevant text:

"Newly emerged and mated G. natalensis females were found to have a pre-oviposition period of two to three days during which they remained either inside the lid of the rearing jar or on the sides of the sugarcane pieces. After this period they were seemingly attracted to the frass produced by their potential host from its boring. The parasitoid entered the boring and stung the larva, temporarily paralysing it, whereupon oviposition commenced."

"The eggs, about 0,6mm long and 0,2mm wide, were laid transversely across the larvae, close to the intersegmental folds (Fig. 2.)"

Table 2. legend says means and 95% CLs given: "Mean progeny per successful female G. natalensis [under different rearing conditions]: 5,2 (0,6)......7,4 (2,8)....9,0(1,1)...."

"The G. natalensis larvae hatched after about three days, pierced the intersegmental membrane of their host, and started feeding (Fig. 5). They continued feeding with their mouth parts and first few larval segments inside the haemocoel of the host (Fig. 6). After 10 to 14 days the larvae withdrew from the host before spinning a cocoon and pupating (Fig. 7). The pupal period lasted another 10 to 14 days. During the larval period the female parent stayed in the boring with her offspring."

"The males lived for a maximum of 11 days (mean = 6,1 ± 2,8; n=30), and the females for a maximum of 28 days (mean = 13,1 ± 1,26; n = 500). "

Example 134

Taxonomy: Hymenoptera: Bethylidae: *Parascleroderma berlandi*

Record type: literature

Life stage(s): egg, adult

Source: Maneval (1930)

Relevant text:

"En taillant l'écorce avec précaution je mis à jour une fente horizontalle très irrégulière, faisant suite à la galerie vertical suivie par la bête, continuée en tous sens par des craquelures zigzaguées, et présentant une loge naturelle de 1 cm. de haut sur 5 mm. de large, nullement aménagée (fig. 3, d). C'est là que reposait la proie, placée la tête en haut et portant un oeuf. Auncune cloison ne l'enfermait et le chasseur retiré dans la galerie horizontale paraissait avour terminé son travail. La proie était cette fois encore une jeune larva de Thanasimus formicarius, un peu plus grande que la précédente, bien paralysée mais présentant néanmoins quelques mouvements limités des pattes et de faibles contractions de l'arrière-corps. L'oeuf était fixé longitudinalement sur le pli latéral des segments pro- et méso-thoraciques, son pôle céphalique tourné vers l'arrière de la proie (Fig. 3, a). Vu en regardant celle-ci de trois quarts, son profil accuse une certaine courbure (fig. 2, c). Il est d'un blanc laiteux, translucide, arrondi au deux bouts et mesure 0,75 mm. de long sur 0,20 mm. de large."

English translation, courtesy of Google translate:

"Trimming the bark with care I uncovered a very irregular horizontal slit, following the vertical gallery followed by the beast, continued in all directions by zigzagged cracks, and having a natural box of 1 cm. high on 5 mm. wide, undeveloped (Fig. 3, d). This is where the prey rested, placed upside down and carrying an egg. A wall did not shut him up, and the hunter, withdrawn into the horizontal gallery, seemed to have finished his work. The prey was once again a young larva of Thanasimus formicarius, a little larger than the previous one, well paralyzed but nevertheless presenting some limited movements of the legs and weak contractions of the hindquarters. The egg was fixed longitudinally on the lateral fold of the pro and meso-thoracic segments, with its cephalic pole turned towards the rear of the prey (Fig. 3, a). Seen looking at it three-quarters, his profile shows a certain curvature (Figure 2, c). It is milky white, translucent, rounded at both ends and measures 0.75 mm. long on 0,20 mm. wide."

Example 135

Taxonomy: Hymenoptera: Bethylidae: *Sclerodermus manoa*

Record type: literature

Life stage(s): egg, larva, adult

Source: Bridwell (1919)

Relevant text:

"This Sclerodermus was found in a cavity in a small stub of white rotten wood, probably the remains of a bush of Scaevola chamissoniana along the Monoa cliffs trail in the mountains back of Honolulu on May 26, 1918. It was there associated with a limp immobile lepidopterous larva supposed to be that of a species of Semnoprepia. The Sclerodermus and the caterpillar were placed in a glass tube and brought in for observation. On the next day she had laid five eggs scattered about on the glass of the tube...

On May 29, only four of the live eggs could be accounted for. One had hatched and the larva was attached to the Semnoprepia larva and this one alone of the progeny reached full larval growth and it failed to spin its coccoon and transform."

"It is evident that sucking the juices of the larval prey is her means of subsistence. By June 13 three eggs had been laid. The larva from the former lot of eggs which reached full growth failed to spin a coccoon or pupate. The female remained alive from May 26, when she was taken, until June 29 feeding readily upon the juices of any caterpillar given her but ovipositing only upon her natural prey."

Example 136

Taxonomy: Hymenoptera: Braconidae: *Adelura subcompressa*
Record type: specimen
Life stage(s): adult
Source: iDigBio
Record URL: https://www.idigbio.org/portal/records/9b5b89ff-0318-43e4-aa75-12f548a8d528
Label image: http://collections.nmnh.si.edu/media/index.php?irn=10517368
Relevant label text:

"observed ovipositing on dipterous larvae in rotten fungus"

Example 137

Taxonomy: Hymenoptera: Braconidae: *Alphomelon nanosoma*
Record type: specimen
Life stage(s): adult
Source: iDigBio
Record URL: https://www.idigbio.org/portal/records/fb7d7803-00ab-44db-bda4-edd906697c24
Label image: http://collections.nmnh.si.edu/media/index.php?irn=10515473
Relevant label text:

"host: Cobalopsis sp. nr. ff. (Hesperiidae) | plant: Olyra latifolia (Poaceae)"

Example 138

Taxonomy: Hymenoptera: Braconidae: *Alphomelon xestopyga*
Record type: specimen
Life stage(s): adult
Source: iDigBio
Record URL: https://www.idigbio.org/portal/records/02c95b73-3f0f-40c6-ad17-8928f33f4ba7
Label image: http://collections.nmnh.si.edu/media/index.php?irn=10515523
Relevant label text:

"host: Calpodes ethlius (Hesperiidae) | plant: Marantha arundinacea (Marantaceae)"

Example 139

Taxonomy: Hymenoptera: Braconidae: *Apanteles absonus*
Record type: specimen
Life stage(s): adult
Source: iDigBio
Record URL: https://www.idigbio.org/portal/records/2aafcb23-1421-4426-a7ab-be0ed9a8f4c1
Label image: http://collections.nmnh.si.edu/media/index.php?irn=10518574
Relevant label text:

"ex. Choristoneura fumiferana"

Example 140

Taxonomy: Hymenoptera: Braconidae: *Apanteles acrobasidis*
Record type: specimen
Life stage(s): adult
Source: iDigBio
Record URL: https://www.idigbio.org/portal/records/44521432-bfe6-46ba-96d9-10d41745eff6
Label image: http://collections.nmnh.si.edu/media/index.php?irn=10518598
Relevant label text:

"ex Acrobasis caryae on English walnut"

Example 141

Taxonomy: Hymenoptera: Braconidae: *Apanteles alius*
Record type: specimen
Life stage(s): adult
Source: iDigBio
Record URL: https://www.idigbio.org/portal/records/afb651b0-25e5-46be-a4da-a140731a36e6
Label image: http://collections.nmnh.si.edu/media/index.php?irn=10518666
Relevant label text:

"ex Opsiphanes on Musa sapientum"

Example 142

Taxonomy: Hymenoptera: Braconidae: *Apanteles baldufi*
Record type: specimen
Life stage(s): adult
Source: iDigBio
Record URL: https://www.idigbio.org/portal/records/17cb1a77-a90d-4c53-bbde-3f4d5d12bed4
Label image: http://collections.nmnh.si.edu/media/index.php?irn=10518844
Relevant label text:

"ex Acrobasis rubrifasciella | reared by W. V. Balduf"

Example 143

Taxonomy: Hymenoptera: Braconidae: *Apanteles californicus*
Record type: specimen
Life stage(s): adult
Source: iDigBio
Record URL: https://www.idigbio.org/portal/records/a62714ba-858a-45da-a05a-5c03e0ec69ec
Label image: http://collections.nmnh.si.edu/media/index.php?irn=10518974
Relevant label text:

"reared | Pinus murrayana"

Example 144

Taxonomy: Hymenoptera: Ceraphronidae: *Aphanogmus* sp.

Record type: literature

Life stage(s): pupa

Source: Luhman et al. (1999)

Relevant text:

"Pupae of the microcaddisfly Ochrotrichia moselyi Flint (Trichoptera: Hydroptilidae) were collected in Costa Rica that contained pupae of Aphanogmus sp. (Hymenoptera: Ceraphronidae). The caddisflies were collected in Puntarenas Province, Bellavista River, ca. 1.5 km NW of Las Alturas, at 1400 m elevation (8.95rN, 82.846°W). Collections were made June 15-17, 1986. All material is housed in the University of Minnesota Insect Collection, St. Paul, Minnesota. Three collections in alcohol yielded one Aphanogmus pupa each. There were a total of 12 Ochrotrichia moselyi cocoons of which 3 contained Aphanogmus pupae, 6 contained eaten caddisfly pupae, and the remainder, developed caddisflies. The cocoons with the parasitoid pupae contained only the wings and cast larval skin of the caddisfly. There was one Aphanogmus per parasitized cocoon. Dr Paul Dessart, a ceraphronid specialist in Belgium, confirmed Luhman's identifications of the Aphanogmus in the cocoons and stated this to be a new ordinal and family host record for Ceraphronidae (pers. comm., 1996). Heretofore recorded hosts of Ceraphronidae included Diptera, Homoptera, Hymenoptera, Neuroptera, and Thysanoptera (Muesebeck 1979; Hanson and Cauld 1995). The Aphanogmus were discovered inside the cocoons of Ochrotichia moselyi, but outside of the caddisfly pupa. Only the developed wings remained uneaten. The Ochrotichia cocoons may have been parasitized when exposed to the surface near the water substrate interface. Ochrotichia pupate within their larval cases in small clusters of individuals on the sides of rocks and boulders. During the dry season, pupae are often exposed or closer to the surface."

Peter Mayhew: Below four examples of life history data from bethylid wasps (Hymenoptera: Bethylidae). Data I wanted for this study were clutch or brood sizes, brood guarding behaviour, egg size, development times, adult longevity, adult body length. I have a collection of many hundreds of papers like this which I used to data mine parasitoid wasp life history traits, now extending this to other insect groups.Examples of useful text (useful data in bold):

Example 145

Taxonomy: Hymenoptera: Formicidae: *Camponotus chromaiodes*

Record type: specimen

Life stage(s): unknown

Source: Johnson et al. (2018)

Record URL: https://hol.osu.edu/spmInfo.html?id=OSAL%200101212

Comments and relevant label text:

Specimen is a mite (Mesostigmata: Oplitidae).

"phoretic on Camponotus chromaiodes Bolton"

"Woods, 613 Smokey Hollow Rd., Piketon, Pike Co., OH, U.S.A. | Uppstrom, K | 21-Sep-2009 | forest | in large stump | ex Camponotus chromaiodes worker | gaster near acidophore] [publication voucher | KAU-09-0910-8 #14 | Oplitis sp. 3"

Collection: Ohio State University Acarology Laboratory, Columbus, OH (OSAL)

Example 146

Taxonomy: Hymenoptera: Ichneumonidae: *Acrodactyla degener*
Record type: specimen
Life stage(s): adult
Source: Natural History Museum (2014)
Record URL: http://data.nhm.ac.uk/object/24c1002b-874f-4051-949c-49e2f570156c
Relevant label text:

"Rothamsted light trap"

Example 147

Taxonomy: Hymenoptera: Ichneumonidae: *Diacritus aciculatus*
Record type: specimen
Life stage(s): adult
Source: Natural History Museum (2014)
Record URL: http://data.nhm.ac.uk/object/f2287913-53ce-46cd-86b0-739f4fcf8cf2
Relevant label text:

"mercury vapour light trap"

Example 148

Taxonomy: Hymenoptera: Ichneumonidae: *Ephialtes manifestator*
Record type: specimen
Life stage(s): adult
Source: Natural History Museum (2014)
Record URL: http://data.nhm.ac.uk/object/ce0997c1-62c5-4a19-8867-bc0fc7fbc2cc
Relevant label text:

"Rothamsted light trap"

Example 149

Taxonomy: Hymenoptera: Platygastridae: *Platygaster chilophagae*
Record type: specimen
Life stage(s): adult
Source: Johnson et al. (2018)
Record URL: https://hol.osu.edu/spmInfo.html?id=USNMENT01059053
Comments and relevant label text:

"SOUTH DAKOTA: Brookings Co. Aurora Research Farm, 4 mi. E. of Brookings, coll. 11.vii.2012, em. 16.vii.2012 from Chilophaga virgati; V. Calles Torrez | Platygaster chilophagae P.N. Buhl det. 2012 | HOLOTYPE P.N.BUHL"

Collection: National Museum of Natural History (USNM)

Example 150

Taxonomy: Hymenoptera: Platygastridae: *Tetrabaeus americanus*

Record type: specimen

Life stage(s): adult

Source: Johnson et al. (2018)

Record URL: https://hol.osu.edu/spmInfo.html?id=USNM%20Type%20No.%2066599

Comments and relevant label text:

"emerged from Ectemnius paucimaculatus (Packard)"

Collection: National Museum of Natural History (USNM)

Example 151

Taxonomy: Hymenoptera: Platygastridae: *Tetrabaeus americanus*

Record type: literature

Life stage(s): larva, adult

Source: Muesebeck (1963)

Relevant text:

"Described from 150 females and 29 males, all from Plummers Island, Maryland, and all reared in 1961 and 1962 by K. V. Krombein from isolated larvae of the crabronine wasps, Euplilis rufigaster (Packard), Euplilis sp. and Ectemnius paucimaculatus (Packard). Both the holotype and the allotype were reared from E. paucimaculatus on September 18, 1962.

This is a gregarious internal parasite. As many as 26 adults have been reared from a single wasp larva. The parasitic larvae completely consume the contents of the host, leaving only the head capsule and the transparent skin through which the parasites are clearly visible."

"the ratio of males to females is only 1 to 5 in the material thus far examined"

One of the specimens referenced in this publication is in the USNM, see the example with the URL https://hol.osu.edu/spmInfo.html?id=USNM%20Type%20No.%2066599.

Example 152

Taxonomy: Hymenoptera: Pompilidae: *Anoplius semirufus*

Record type: specimen

Life stage(s): adult

Source: iDigBio

Record URL: https://www.idigbio.org/portal/records/789f5a99-df20-4260-9052-36a89ffa36e8

Label image: https://api.idigbio.org/v2/media/049063a37a2fd7dd27346a8b839f352f?size=fullsize

Relevant label text:

"feeding at flower of Paronychia chartacea"

Example 153

Taxonomy: Hymenoptera: Sapygidae: *Fedtschenkia anthracina*

Record type: literature

Life stage(s): adult

Source: Bohart and Schuster (1972)

Relevant text:

"In 1956 at Tanbark Flat in the San Gabriel mountains of California the senior author observed a female Fedtschenkia anthracina (Ashmead) entering a ground burrow which was found to contain a cell with a dead adult of the eumenid wasp, Pterocheilus trichogaster R. Bohart. Evidence of a relationship seemed flimsy until D.J. Horning and the junior author excavated a number of burrows of P. trichogaster on Santa Cruz Island, California late in April of 1969. Fedtschenkia were abundant and active as parasites in the nesting area. Finally, the senior author at Arroyo Seco, Monterey County, California in May 1971 observed F. anthracina females entering and staying for considerable periods in burrows of P. trichogaster which were provisioning with geometrids of the genus Hydriomena Hübner (det. M.R. Gardner)."

Example 154

Taxonomy: Hymenoptera: Scelionidae: *Gryon aculeator*

Record type: specimen

Life stage(s): adult

Source: Johnson et al. (2018)

Record URL: https://hol.osu.edu/spmInfo.html?id=USNMENT01059225

Comments and relevant label text:

"Parasite of strawberry leafroller | Blair, Ks. July 1937 | P. G. Lamerson Coll. | HOLOTYPE Gryon aculeator Masner"

Collection: National Museum of Natural History (USNM)

Example 155

Taxonomy: Hymenoptera: Scelionidae: *Gryon anasae*

Record type: specimen

Life stage(s): adult

Source: Johnson et al. (2018)

Record URL: https://hol.osu.edu/spmInfo.html?id=USNMENT00979994

Comments and relevant label text:

"Jacksnville Fla | Collection Ashmead | Ex Anasa tristis eggs | Type No. 2852 U.S.N.M. | Hadronotus (Telenomus) anasae Type Ashm | Holotypus Hadronotus anasae Ash. G. MINEO, 1985 severed | LECTOTYPE Hadronotus anasae Ashmead Masner & Muesebeck, 1968"

This is from the publication: "from May 15 to August 31, 1886" and "bred in June and July".

Collection: National Museum of Natural History (USNM)

Example 156

Taxonomy: Hymenoptera: Scelionidae: *Gryon chelinideae*
Record type: specimen
Life stage(s): adult
Source: Johnson et al. (2018)
Record URL: https://hol.osu.edu/spmInfo.html?id=USNMENT01059234
Comments and relevant label text:

"Ex eggs Chelinidea sp. | Uvalde Tex | RCMundell coll. ix.14'30 | 6252Hm | HOLOTYPE Gryon chelinidae MSN. CNC No.

Collection: National Museum of Natural History (USNM)

Example 157

Taxonomy: Hymenoptera: Scelionidae: *Gryon leptocorisae*
Record type: specimen
Life stage(s): adult
Source: Johnson et al. (2018)
Record URL: https://hol.osu.edu/spmInfo.html?id=USNMENT00989860
Comments and relevant label text:

"Ex eggs of Stenocoris tipuloides | Crescent City Fla | 44C"

Collection: National Museum of Natural History (USNM)

Example 158

Taxonomy: Hymenoptera: Scelionidae: *Gryon myrmecophilum*
Record type: specimen
Life stage(s): adult
Source: Johnson et al. (2018)
Record URL: https://hol.osu.edu/spmInfo.html?id=USNMENT00989861
Comments and relevant label text:

"Arlington Va | Type | Type No. 21925 U.S.N.M."

From publication: "taken from an ant's nest".

Collection: National Museum of Natural History (USNM)

Example 159

Taxonomy: Hymenoptera: Scelionidae: *Trissolcus japonicus*
Record type: specimen
Life stage(s): adult
Source: Johnson et al. (2018)
Record URL: https://hol.osu.edu/spmInfo.html?id=USNMENT01059400
Comments and relevant label text:

"USA: Maryland: Beltsville 39°01’42"N, 76°55’47"W woods, coll. M. Herlihy | reared from fresh sentinel BMSB [brown marmorated stink bug] eggs, 72 hr exp, ending 2.IX.2014 #492 | Trissolcus japonicus det. Talamas 2015"

Collection: National Museum of Natural History (USNM)

Example 160

Taxonomy: Hymenoptera: Scelionidae: *Trissolcus japonicus*

Record type: specimen

Life stage(s): adult

Source: Johnson et al. (2018)

Record URL: https://hol.osu.edu/spmInfo.html?id=USNMENT01081080

Comments and relevant label text:

"Winchester, VA June 15, 2015 J.C. Bergh | from sentinel BMSB [brown marmorated stink bug] eggs 6-15 #1a | Collecting dates reflect the dates which the sentinel eggs (fresh) of BMSB were deployed."

"emerged from egg of Halyomorpha halys (Stål), collected on Ailanthus altissima (Mill.) Swingle"

Collection: National Museum of Natural History (USNM)

Example 161

Taxonomy: Hymenoptera: Scelionidae: *Trissolcus plautiae*

Record type: specimen

Life stage(s): adult

Source: Johnson et al. (2018)

Record URL: https://hol.osu.edu/spmInfo.html?id=USNMENT00916874

Comments and relevant label text:

"CHINA: Beijing Prov Sheng shuyuan 40°3'13"N 116°7'9 | ex. eggs Plautia fimbriata on mulberry Coll. Tim Haye, 1.VI.2013 P167"

"collected on mulberry, emerged from egg of Plautia fimbriata (Fabricius)"

Collection: National Museum of Natural History (USNM)

Example 162

Taxonomy: Hymenoptera: Scelionidae: *Trissolcus solocis*

Record type: specimen

Life stage(s): adult

Source: Johnson et al. (2018)

Record URL: https://hol.osu.edu/spmInfo.html?id=DPI_FSCA+00009872

Comments and relevant label text:

"USA: AL: Prattville, coll. Tillman 23-IX-2017 ex. frozen BMSB [brown marmorated stink bug] eggs on cotton"

"collected on cotton, emerged from egg of Halyomorpha halys (Stål)"

"reared from frozen sentinel egg mass"

Collection: Florida State Collection of Arthropods (FSCA)

Example 163

Taxonomy: Hymenoptera: Torymidae: *Megastigmus helinae*

Record type: literature

Life stage(s): adult

Source: Roques et al. (2016)

Relevant text:

"Megastigmus helinae Roques & Copeland, sp. n. was reared from seeds of the Rhamnaceae Helinus integrifolius (Lam.) Kuntze (Figure 10), collected in the northern Kenya mountains of the Mathews Range."

"Holotype ♀, Kenya, Scandent climber, Rift Valley Prov. Matthews Range, 1.1777°N, 37.3141°E, 1342m, 16 Jan 2004, ex. Helinus integrifolius fruits, A&M coll. N°2692, R. Copeland leg. (NMKE)."

"Briefly, fruits were collected from plants or on the ground below them. An effort was made to collect ripe, but not rotting fruit."

"In the laboratory, fruits were removed from transport bags and placed within one-litre, rectangular plastic containers that had small elliptical holes cut out of the bottom. Each one-litre container (also provided with a mesh-covered lid) was nested within a fresh two-litre container, the bottom of which had a layer of heat-treated sand. Fruits were usually held for up to two months. Emerged adult insects were held for 1–3 days before being killed."

Lepidoptera

Example 164

Taxonomy: Lepidoptera: Epipyropidae: *Fulgoraecia melanoleuca*

Record type: literature

Life stage(s): larva, pupa

Source: Kumar et al. (2015)

Relevant text:

"Larva removed from the abdomen of Pyrilla perpusilla", "100 pupae, sorghum and wheat", "50 larvae on Pyrilla perpusilla, Sorghum, IARI, New Delhi, 20.03.2008"

Example 165

Taxonomy: Lepidoptera: Erebidae: *Euceron leucophacum*

Record type: specimen

Life stage(s): adult

Source: MGCL

Comments and relevant label text:

Specimen ID: LEP-38704

"Zapped-Sound producer": Insect was held in place and exposed to recordings of bat ultrasound to determine whether the insect would produce ultrasound in response. In this case, the moth did produce ultrasound in response.

Example 166

Taxonomy: Lepidoptera: Erebidae: *Lophocampa caryae*

Record type: specimen

Life stage(s): larva

Source: iDigBio

Record URL: https://www.idigbio.org/portal/records/31c6c108-41a9-4802-86d2-fc31ad387b6c

Label image: http://scan-bugs.org/imglib/scan/MSU_MSUC/201606/Arctiidae17B-Lophocampa_caryae_1466017617_lg.jpg

Relevant label text:

"KAAD to 75% EtOH", "ex. trees"

Example 167

Taxonomy: Lepidoptera: Gelechiidae: *Ephysteris kullbergi*

Record type: literature

Life stage(s): adult

Source: Bidzilya and Karsholt (2018)

Relevant text:

"♂", "RUSSIA Tuva rep. 50°01’N, 95°03’E, 1150 m, Lake Tere-Khol, sand dunes 9.–12.6.1995 Jalava & Kullberg leg." "genitalia slide 153/16, O. Bidzilya" (MZH)

Example 168

Taxonomy: Lepidoptera: Hepialidae: *Phassus huebneri*

Record type: specimen

Life stage(s): egg

Source: MGCL

Comments and relevant label text:

Specimen ID: LEP-58836

"Eggs of LEP-58835"

Example 169

Taxonomy: Lepidoptera: Lasiocampidae: *Malacosoma californicum lutescens*

Record type: specimen

Life stage(s): larva

Source: iDigBio

Record URL: https://www.idigbio.org/portal/records/cb047dca-f460-4aeb-9770-17a8dd94bc29

Label image: https://api.idigbio.org/v2/media/a7959b205b3faa745e5205de6fbc5fe2?size=fullsize

Relevant label text:

"Host: Wild plum"

Example 170

Taxonomy: Lepidoptera: Lasiocampidae: *Tolype* sp.

Record type: specimen

Life stage(s): larva

Source: MGCL

Comments and relevant label text:

Specimen ID: LEP-46601

"larvae, Host plant Gossypilam Harleri"

Example 171

Taxonomy: Lepidoptera: Lycaenidae

Record type: literature

Life stage(s): larva

Source: Wiklund (1984)

Relevant text:

"That is, butterflies that overwinter in the egg stage tend not to deposit their eggs on the larval host plants. The main exception to this pattern is furnished by butterflies that use woody perennials as host plants, viz. the hairstreaks Thecla betulae, Quercusia quercus, Strymonidia w-album, and the blues Plebejus argus and Lycaeides idas. All of the eight Swedish species that I have studied and that use herbaceous host plants, viz. Parnassius apollo, P. mnemosyne, Argynnis paphia, Mesoacidalia aglaja, Fabriciana adippe, F. niobe, Erebia ligea, Heodes virgaureae and Aricia nicias, avoid depositing their eggs on the green parts of their host plants (Table 1). Of these eight species, all but two clearly locate their larval host plants first, but then displace themselves some distance from the host plant before depositing the egg. A. nicias seems to be a special case as it actually deposits the eggs on the leaves of the host plant, but almost invariably lays the eggs on leaves that are completely wilted and dry."

Example 172

Taxonomy: Lepidoptera: Lycaenidae: *Callophrys irus*

Record type: specimen

Life stage(s): adult

Source: iDigBio

Record URL: https://www.idigbio.org/portal/records/3542e16c-8c7e-4a77-b365-be19c6a11c6a

Label image: http://scan-bugs.org/imglib/scan/VPI_VTEC/VTEC000000/VTEC000000439_1483998384_lg.jpg

Relevant label text:

"♂"

Example 173

Taxonomy: Lepidoptera: Lycaenidae: *Cyclargus thomasi*

Record type: specimen

Life stage(s): adult

Source: MGCL

Comments and relevant label text:

Specimen ID: LEP-48516

"Reared, body dry in tube, Lab reared, ex. wild caught female 19.XI.2017"

Example 174

Taxonomy: Lepidoptera: Nymphalidae: *Anaea andria*

Record type: specimen

Life stage(s): adult

Source: iDigBio

Record URL: https://www.idigbio.org/portal/records/9fcc1c51-28e1-4079-901b-c191d4b0c648

Label image: https://s.idigbio.org/idigbio-images-prod-fullsize/dbcd49d51b73226c44ea71c9a0985bb6.jpg

Relevant label text:

"♂"

Example 175

Taxonomy: Lepidoptera: Papilionidae: *Papilio cresphontes*

Record type: specimen

Life stage(s): larva

Source: iDigBio

Record URL: https://www.idigbio.org/portal/records/92d1232d-5601-4434-bcaa-50a354657074

Label image: https://api.idigbio.org/v2/media/bdff1d0477d19fa28e23a815c7181826?size=fullsize

Relevant label text:

"feeding on wild cherry"

Example 176

Taxonomy: Lepidoptera: Pyralidae: *Basacallis tarachodes*

Record type: literature

Life stage(s): larva, pupa, adult

Source: Hayden et al. (2017)

Relevant text:

"In the laboratory, KS found lepidopteran larvae in silken tubes in the soil (Fig. 7–8)."

"JH preserved some larvae (Fig. 11) and found the gut contents to be soil from the latrine [of a rodent in a small cave]."

"JH separated the larvae individually into small plastic cups and collection vials, ventilated with holes and supplied with enough of the original soil to tunnel in. Larvae were inspected every two or three days. Starting on 29 December 2015, JH moistened the soil with a few drops of purified water every few days. By 4 January 2016, larvae were found in ten containers and absent from four; no bodies were found. Between 4 and 7 January, three more pupated (Fig. 12). Two adults eclosed on 9 and 10 January (Fig. 14), 2 on 11 Jan., 3 by 13 Jan., and a fourth on 15 January."

"A few larvae were found in additional, moderately fresh scat."

Example 177

Taxonomy: Lepidoptera: Pyralidae: *Eldana saccharina*

Record type: literature

Life stage(s): larva, pupa

Source: Conling et al. (1988)

Relevant text:

"In Cyperus papyrus, the larval and pupal stages of E. saccharina are found in the umbels at the base of the bracts (the apical end of the culm) and in the rhizomes, when these are not covered by water."

Example 178

Taxonomy: Lepidoptera: Saturniidae: *Actias luna*

Record type: specimen

Life stage(s): larva (5th instar?)

Source: iDigBio

Record URL: https://www.idigbio.org/portal/records/bf1f9d1c-d7e9-4f77-9391-d1692526dc4b

Label image: https://api.idigbio.org/v2/media/2b0e11776c0f49e38cbe069ea1f4d910?size=fullsize

Relevant label text:

"ova from captured female | larvae reared on walnut & hickory", "KAAD --> 75% EtOH"

Example 179

Taxonomy: Lepidoptera: Saturniidae: *Anisota pellucida*

Record type: specimen

Life stage(s): adult

Source: MGCL

Comments and relevant label text:

Specimen ID: LEP-51550

"male / Ex. ova from captive female paired with wild Gainesville male, reared by R. St Laurent"

Example 180

Taxonomy: Lepidoptera: Saturniidae: *Automeris io*

Record type: specimen

Life stage(s): larva

Source: MGCL

Comments and relevant label text:

Specimen ID: LEP-60503

"Reared for D. Triant Genome Project; Frozen in Liquid Nitrogen; 2016-10 Sugarberry 6th Instar"

Example 181

Taxonomy: Lepidoptera: Saturniidae: *Citheronia sepulcralis*

Record type: specimen

Life stage(s): adult

Source: MGCL

Comments and relevant label text:

Specimen ID: LEP-44917

"Ex. ova from female collected by D. Halbritter, reared by R. St Laurent on Pinus taeda. Eclosed 14.XII.2016"

Example 182

Taxonomy: Lepidoptera: Saturniidae: *Dirphia avia*

Record type: specimen

Life stage(s): adult

Source: MGCL

Comments and relevant label text:

Specimen ID: LEP-38854

"Zapped-Sound Producer, Bat, Tactile": Insect was held in place and exposed to recordings of bat ultrasound to determine whether the insect would produce ultrasound in response. In this case, the moth did not produce ultrasound in response. "Tactile" indicates that the moth also produced ultrasound in response to being touched.

Example 183

Taxonomy: Lepidoptera: Saturniidae: *Eacles imperialis imperialis*

Record type: specimen

Life stage(s): larva ("middle instar")

Source: iDigBio

Record URL: https://www.idigbio.org/portal/records/e2cb7707-43cd-4c32-99b9-ce24514c1dd7

Label image: https://api.idigbio.org/v2/media/f516c38f23b237a5f1af0a7d335e4670?size=fullsize

Relevant label text:

"ex. female at lights | ex. ova | larva reared on oak – Quercus sp. | larva killed/preserved 3 August 2010"

Example 184

Taxonomy: Lepidoptera: Saturniidae: *Eacles imperialis pini*

Record type: specimen

Life stage(s): larva (3rd instar?)

Source: iDigBio

Record URL: https://www.idigbio.org/portal/records/1f30e210-2a11-402e-9763-498be43d8dee

Label image: https://api.idigbio.org/v2/media/6472d1bc4595acbc9dd83de0aaba7efb?size=fullsize

Relevant label text:

"x -ova- larva fed on jack pine"

Example 185

Taxonomy: Lepidoptera: Saturniidae: *Psilopygoides oda*

Record type: specimen

Life stage(s): adult

Source: MGCL

Comments and relevant label text:

Specimen ID: LEP-34861

"zapped negative": Insect was held in place and exposed to recordings of bat ultrasound to determine whether the insect would produce ultrasound in response. In this case, the moth did not produce ultrasound in response.

Example 186

Taxonomy: Lepidoptera: Sesiidae: *Bembecia marginatum*

Record type: specimen

Life stage(s): larva, pupa

Source: iDigBio

Record URL: https://www.idigbio.org/portal/records/8cbd3423-d15e-42a0-88f2-ecf4d91637ce

Label image: https://api.idigbio.org/v2/media/30f0bad6461f42d55edc15bd63e7a45a?size=fullsize

Relevant label text:

"Crown of raspberry", "KAAD --> 75% EtOH"

Example 187

Taxonomy: Lepidoptera: Sphingidae: *Adhemarius gannascus*

Record type: specimen

Life stage(s): adult

Source: MGCL

Comments and relevant label text:

Specimen ID: LEP-42646

"Emerged 11/5/2016 (reared)"

Example 188

Taxonomy: Lepidoptera: Sphingidae: *Hippotion* sp.
Record type: specimen
Life stage(s): adult
Source: MGCL
Comments and relevant label text:

Specimen ID: LEP-63008

"Sound producing": The moth produced ultrasound in response to playbacks of bat recordings, physical touch, or both.

Example 189

Taxonomy: Lepidoptera: Uraniidae: *Urania* sp.
Record type: specimen
Life stage(s): adult
Source: MGCL
Comments and relevant label text:

Specimen ID: LEP-59094

"Day Flying Moth"

References

Akre, R.D., G. Alpert, T. Alpert (1973) Life cycle and behavior of *Microdon cothurnatus* in Washington (Diptera: Syrphidae). *Journal of the Kansas Entomological Society* 46: 327-338. http://www.jstor.org/stable/25082580

Aldrich, J.M. (1916) *Sarcophaga and Allies in North America*. Lafayette, IN: Murphey-Bivins Co. Press. 301 pp.

Anderson, G.S., S.L. VanLaerhoven (1996) Initial studies on insect succession on carrion in southwestern British Columbia. *Journal of Forensic Sciences* 41: 13964J. DOI: 10.1520/JFS13964J

Atkinson, T.H. Bark and Ambrosia Beetles Database. http://barkbeetles.info/projects_database.php

Benda, N., J. Possley, D. Powell, C. Buchanan-Mcgrath, J. Cuda (2012) New host plant record for the poison ivy sawfly, *Arge humeralis* (Hymenoptera: Argidae), and its performance on two host plant species. *Florida Entomologist* 95: 529-531. DOI: 10.1653/024.095.0248

Bidzilya, O., O. Karsholt (2018) Two new species of *Ephysteris* Meyrick, 1908, from Asia with brachypterous males (Lepidoptera, Gelechiidae). *Nota Lepidopterologica* 41: 107-112. DOI: 10.3897/nl.41.23395

Bohart, R., R. Schuster (1972) A host record for *Fedtschenkia* (Hymenoptera: Sapygidae). *Pan-Pacific Entomologist* 48: 149. https://biodiversitylibrary.org/page/53786590

Bridwell, J. (1919) Some notes on Hawaiian and other Bethylidae (Hymenoptera) with descriptions of new species. *Proceedings of the Hawaiian Entomological Society* 4: 21-38. https://www.biodiversitylibrary.org/item/39842#page/57/mode/1up

Carlton, C., V. Bayless (2011) A case of *Cnestus mutilatus* (Blandford) (Curculionidae: Scolytinae: Xyleborini) females damaging plastic fuel storage containers in Louisiana, U.S.A. *The Coleopterists Bulletin* 65: 290-291. DOI: 10.1649/072.065.0308

Charlwood, J., J. Pinto, C. Sousa, H. Madsen, C. Ferreira, V. do Rosario (2002) The swarming and mating behaviour of *Anopheles gambiae* s.s. (Diptera: Culicidae) from São Tomé Island. *Journal of Vector Ecology* 27: 178-183. http://www.sove.org/Journal/Entries/2002/12/1_Volume_27,_Number_2_files/Charlwood.pdf

Chicago Department of Public Health (2018) West Nile Virus Mosquito Test Results. https://data.cityofchicago.org/Health-Human-Services/West-Nile-Virus-WNV-Mosquito-Test-Results/jqe8-8r6s

CNC: Canadian National Collection of Insects, Arachnids, and Nematodes. Ottawa, Ontario, Canada. http://www.agr.gc.ca/eng/science-and-innovation/agriculture-and-agri-food-research-centres-and-collections/canadian-national-collection-of-insects-arachnids-and-nematodes-cnc/?id=1270047992811

Coffey, M.D. (1966) Studies on the association of flies (Diptera) with dung in southeastern Washington. *Annals of the Entomological Society of America* 59: 207-218. DOI: 10.1093/aesa/59.1.207

Conling, D., D. Graham, H. Hastings (1988) Notes on the natural host surveys and laboratory rearing of *Goniozus natalensis* Gordh (Hymenoptera: Bethylidae), a parasitoid of *Eldana saccharina* Walker (Lepidoptera: Pyralidae) larvae from *Cyperus papyrus* L. in Southern Africa. *Journal of the Entomological Society of Southern Africa* 51: 115-127. https://journals.co.za/content/JESSA/51/1/AJA00128789_2538

Dahlem, G.A., R.F.C. Naczi (2006) Flesh flies (Diptera: Sarcophagidae) associated with North American pitcher plants (Sarraceniaceae), with descriptions of three new species. *Annals of the Entomological Society of America* 99: 218-240. DOI: 10.1603/0013-8746(2006)099[0218:FFDSAW]2.0.CO;2

Danthanarayana, W. (1980) Parasitism of the light brown apple moth, *Epiphyas postvittana* (Walker), by its larval ectoparasite, *Goniozus jacintae* Farrugia (Hymenoptera: Bethylidae), in natural populations in Victoria. *Australian Journal of Zoology* 28: 685. DOI: 10.1071/ZO9800685

Denno, R.F., W.R. Cothran (1976) Competitive interactions and ecological strategies of sarcophagid and calliphorid flies inhabiting rabbit carrion. *Annals of the Entomological Society of America* 69: 109-113. DOI: 10.1093/aesa/69.1.109

Downes, W.L.J. (1965) Family Sarcophagidae. In *A Catalog of the Diptera of America North of Mexico*, pp. 933-961. Washington, D.C.: United States Department of Agriculture.

Faust, L. (2012) Fireflies in the snow: Observations on two early-season arboreal fireflies *Ellychnia corrusca* and *Pyractomena borealis*. *Lampyrid* 2: 48-71. https://www.researchgate.net/profile/Lynn_Faust/publication/280132755_Fireflies_in_the_Snow_Observations_o[illegible] season_arboreal_fireflies_Ellychnia_corrusca_and_Pyractomena_borealis/links/55abd5f308ae481aa7ff4382.pdf

Gottsberger, G. (1989) Beetle pollination and flowering rhythm of *Annona* spp. (Annonaceae) in Brazil. *Plant Systematics and Evolution* 167: 165-187. DOI: 10.1007/BF00936404

Graenicher, S. (1931) Some observations on the biology of the Sarcophaginae (Diptera: Sarcophagidae). *Entomological News* 42: 227-230.

Grassberger, M., C. Frank (2004) Initial study of arthropod succession on pig carrion in a central European urban habitat. *Journal of Medical Entomology* 41: 511-523. DOI: 10.1603/0022-2585-41.3.511

Hall, D.G. (1929) An annotated list of the Sarcophaginae which have been collected in Kansas. *Journal of the Kansas Entomological Society* 2: 83-90. DOI: 10.2307/25081273

Hayden, J.E., L.L. Buss, K.E. Schnepp, L. Xiao (2017) First reports of non-phytophagous Nearctic chrysaugine moths (Lepidoptera: Pyralidae). *Insecta Mundi* 0: 1-8. http://journals.fcla.edu/mundi/article/view/104378

Howard, R.W., D.W. Stanley-Samuelson, R.D. Akre (1990) Biosynthesis and chemical mimicry of cuticular hydrocarbons from the obligate predator, *Microdon albicomatus* Novak (Diptera: Syrphidae) and its ant prey, *Myrmica incompleta* Provancher (Hymenoptera: Formicidae). *Journal of the Kansas Entomological Society* 63: 437-443. http://www.jstor.org/stable/25085201

iDigBio. https://www.idigbio.org/

iNaturalist. https://www.inaturalist.org/

James, M.T. (1947) The flies that cause myiasis in man. *United States Department of Agriculture Miscellaneous Publications* 631: 1-175.

Johnson, N.F., L. Musetti, J. Cora, et al. (2018) Hymenoptera Online database. https://hol.osu.edu/

Knipling, E.F. (1936) A comparative study of the first-instar larvae of the genus *Sarcophaga* (Calliphoridae, Diptera), with notes on the biology. *The Journal of Parasitology* 22: 417. DOI: 10.2307/3271688

Kumar, R., V. Mittal, P. Chutia, V. Ramamurthy (2015) Taxonomy of *Fulgoraecia melanoleuca* (Fletcher, 1939), (Lepidoptera: Epipyropidae) in India, a biological control agent of *Pyrilla perpusilla* (Walker) (Hemiptera: Lophopidae). *Zootaxa* 3974: 431. DOI: 10.11646/zootaxa.3974.3.10

Laake, E., E.C. Cushing, H. Parish (1936) Biology of the primary screw worm fly, *Cochliomyia americana*, and a comparison of its stages with those of *C. macellaria*. *Technical Bulletin, United States Department of Agriculture* 500: 1-24.

Lencioni, V., L. Marziali, B. Rossaro (2012) Chironomids as bioindicators of environmental quality in mountain springs. *Freshwater Science* 31: 525-541. DOI: 10.1899/11-038.1

Lopes, H.d.S. (1988) On Emblemasomini (Diptera, Sarcophagidae), with descriptions of four new species. *Memórias do Instituto Oswaldo Cruz* 83: 17-27. DOI: 10.1590/S0074-02761988000100003

Luhman, J.C., R.W. Holzenthal, J.K. Kjaerandsen (1999) New host record of a ceraphronid (Hymenoptera) in Trichoptera pupae. *Journal of Hymenoptera Research* 8: 126. https://www.biodiversitylibrary.org/page/4490571#page/448/mode/1up

Maneval, H. (1930) Description et moeurs de l'adulte et de la larve d'une espèce nouvelle du genre *Parascleroderma* (Hym. Bethylidae). *Bulletin de la Societe Entomologique de France* 1930: 53-61.

MCZ: Museum of Comparative Zoology. Harvard University, Cambridge, MA, USA. https://mcz.harvard.edu/

MGCL: McGuire Center for Lepidoptera and Biodiversity. Florida Museum of Natural History, University of Florida, Gainesville, FL, USA. https://www.floridamuseum.ufl.edu/index.php/mcguire/home/

Middlekauff, W. (1959) Some biological observations on *Sarcophaga falciformis*, a parasite of grasshoppers (Diptera: Sarcophagidae). *Annals of the Entomological Society of America* 52: 724-728.

Muesebeck, C. (1963) A platygasterid parasite of certain wasp larvae. *Beiträge zur Entomologie* 13: 391-394. https://www.zobodat.at/pdf/Beitraege-zur-Entomologie_13_0391-0394.pdf

Mumcuoglu, K., Y. Braverman (2010) Parasitic and phoretic mites of Diptera in Israel and the Sinai Peninsula, Egypt. *Israel Journal of Earth Sciences* 40: 195-203.

Natural History Museum (2014) Dataset: Collection specimens. Natural History Museum Data Portal (data.nhm.ac.uk). DOI: 10.5519/0002965

Paro, C.M., A. Arab, J. Vasconcellos-Neto (2011) The host-plant range of twig-girdling beetles (Coleoptera: Cerambycidae: Lamiinae: Onciderini) of the Atlantic rainforest in southeastern Brazil. *Journal of Natural History* 45: 1649-1665. DOI: 10.1080/00222933.2011.559601

Raju, A.J.S., V. Ezradanam (2002) Pollination ecology and fruiting behaviour in a monoecious species, *Jatropha curcas* L. (Euphorbiaceae). *Current Science* 83: 1395-1398. https://www.jstor.org/stable/24106968

Roback, S.S. (1954) The evolution and taxonomy of the Sarcophaginae. *Illinois Biological Monographs* 23: 1-181.

Roberts, R.A. (1933) Additional notes on myiasis in rabbits (Dipt.: Calliphoridae, Sarcophagidae). *Entomological News* 44: 157-159.

Roberts, R.A. (1933) Biology of *Brachymeria fonscolombei* (Dufour), a hymenopterous parasite of blowfly larvae. *Technical Bulletin, United States Department of Agriculture* 365: 1-21.

Roques, A., R.S. Copeland, L. Soldati, O. Denux, M. Auger-Rozenberg (2016) Megastigmus seed chalcids (Hymenoptera, Torymidae) radiated much more on Angiosperms than previously considered. I- Description of 8 new species from Kenya, with a key to the females of Eastern and Southern Africa. *ZooKeys* 585: 51-124. DOI: 10.3897/zookeys.585.7503

Sama, G., J. Buse, E. Orbach, A.L.L. Friedman, O. Rittner, V. Chikatunov (2010) A new catalogue of the Cerambycidae (Coleoptera) of Israel with notes on their distribution and host plants. *Munis Entomology & Zoology* 5: 1-51. http://www.cerambycoidea.com/titles/samaalii2010.pdf

Sivinski, J.M., J.E. Lloyd, S.N. Beshers, L.R. Davis, R.G. Sivinski, S.R. Wing, R.T. Sullivan, P.E. Cushing, E. Petersson (1998) A Natural History of *Pleotomodes needhami* Green (Coleoptera: Lampyridae): A firefly symbiont of ants. *The Coleopterists Bulletin* 52: 23-30. https://www.jstor.org/stable/4009318?seq=1#page_scan_tab_contents

Stucky, B.J. (2013) Morphology, bioacoustics, and ecology of *Tibicen neomexicensis* sp. n., a new species of cicada from the Sacramento Mountains in New Mexico, U.S.A. (Hemiptera, Cicadidae, *Tibicen*). *ZooKeys* 337: 49-71. DOI: 10.3897/zookeys.337.5950

Stucky, B.J. (2015) Infection behavior, life history, and host parasitism rates of *Emblemasoma erro* (Diptera: Sarcophagidae), an acoustically hunting parasitoid of the cicada *Tibicen dorsatus* (Hemiptera: Cicadidae). *Zoological Studies* 54: 1-17. DOI: 10.1186/s40555-015-0105-z

Stucky, B.J. (2016) Eavesdropping to find mates: The function of male hearing for a cicada-hunting parasitoid fly, *Emblemasoma erro* (Diptera: Sarcophagidae). *Journal of Insect Science* 16: 68. DOI: 10.1093/jisesa/iew048

Sukchit, M., S. Deowanish, B.A. Butcher (2015) Decomposition stages and carrion insect succession on dressed hanging pig carcasses in Nan province, northern Thailand. *Tropical Natural History* 15: 137–153-137–153. https://tci-thaijo.org/index.php/tnh/article/view/103078

Viviani, V.R., J.W. Hastings, T. Wilson (2002) Two bioluminescent Diptera: the North American *Orfelia fultoni* and the Australian *Arachnocampa flava*. Similar niche, different bioluminescence systems. *Photochemistry and Photobiology* 75: 22. DOI: 10.1562/0031-8655(2002)075<0022:TBDTNA>2.0.CO;2

Webster, R.P., J.D. Sweeney (2016) New records of Helophoridae, Hydrochidae, and Hydrophilidae (Coleoptera) from New Brunswick, Canada. *ZooKeys* 573: 19-30. DOI: 10.3897/zookeys.573.7020

Wikimedia Commons. https://commons.wikimedia.org/

Wiklund, C. (1984) Egg-laying patterns in butterflies in relation to their phenology and the visual apparency and abundance of their host plants. *Oecologia* 63: 23-29. DOI: 10.1007/BF00379780
