# Supplementary material for: Developing a vocabulary and ontology for modeling insect natural history data: example data, use cases, and competency questions
Source: Biodivers Data J. 2019 Mar 13;7:e33303. doi: 10.3897/BDJ.7.e33303 (PMC6426826; doi:10.3897/BDJ.7.e33303)
Supplement: Supplementary material 3 — Ontology competency questions, user domains or groups, and example use cases [file bdj-07-e33303-s003.pdf]

**Supplemental 3: Ontology competency questions, user domains or groups, and example use cases**

This document is supplemental information for Stucky et al. (2019) ____________________.

**Table S1.** Ontology competency questions and relevant ontology concept areas for each question. Key to concept area numbers: **1)** observations and observing processes; **2)** relationships and interactions; **3)** single-organism behaviors; **4)** ontogeny; **5)** organism products and traces; **6)** habitat, locality, and substrates; **7)** positional and spatial information; **8)** weather and climate; **9)** collecting methods; **10)** curation. For more information about these concept areas, see Table 1 in the main article.

| Competency question | Concept area(s) |
|---|---|
| 1. What all is known about the natural history of species *X*? | 2-8 |
| 2. What natural history observations do we have for species *X* in North America? | 2-8 |
| 3. What are the positive, negative, and neutral biotic interactions observed for species of the genus *X*? | 2 |
| 4. Toward which species is species *X* antagonistic? | 2 |
| 5. Given a list of species from some community, how do these species interact with each other? | 2 |
| 6. If species *X* were to become locally extinct, what other species might be impacted? | 2 |
| 7. Which insect species might be conserved if a piece of forest with tree species *X*, *Y*, and *Z* is preserved? | 2 |
| 8. Which species visit flowers of the genus *X*? | 2 |
| 9. Which insect species of the family X are associated with trees? | 2 |
| 10. Which species of the genus *X* have not been found on flowers? | 2 |
| 11. Near which tree species does species *X* engage in courtship behaviors? | 2, 3, 6, 7 |
| 12. Are all occurrences of species *X* associated with plant species *Y*? | 2 |
| 13. Which species of the order X have been observed laying eggs on a plant species on which they have not been observed feeding? | 2 |
| 14. What predator/prey interactions have been observed in state park X? | 1, 2 |
| 15. What specimens are associated with observations of predation by species *X*, and where are they located? | 1, 2, 10 |
| 16. From which host life stages has parasitoid species *X* emerged? | 2, 4 |
| 17. Which species of the genus *X* are parasitized by the species *Y*? | 2 |
| 18. From which species have species of the genus *X* emerged? | 2 |
| 19. Which host associations for the parasitoid species *X* are supported by at least 3 different observers? | 1, 2 |
| 20. Are there parasitoids of hyperparasitoids? | 2 |
| 21. What species of insects have been found in two or more hosts from different taxonomic orders? | 2 |
| 22. On eggs of which species has parasitoid species *X* been found? | 2 |
| 23. What does species *X* eat? | 2 |

| Competency question | Concept area(s) |
|---|---|
| 24. What are all the food sources for lab rearing of species *X*? | 2, 4 |
| 25. What predators eat species *X*? | 2 |
| 26. Does species *X* eat carrion? | 2 |
| 27. Do insects ever eat hummingbirds? | 2 |
| 28. Which species farm fungus *X*? | 2 |
| 29. Where does species *X* lay its eggs? | 2, 6, 7 |
| 30. When does species *X* lay its eggs? | 1, 8 |
| 31. Which insects are found on insects that are found on carrion? | 2, 6, 7 |
| 32. Which species of the family X have larvae associated with species of the family Y? | 2 |
| 33. On which parts of the human body have bot flies been found? | 2, 6, 7 |
| 34. Which ant species are known to sting humans? | 2 |
| 35. What are the northern-most records of insects feeding on humans? | 1, 2 |
| 36. Which species of the order X have been collected in houses in Florida? | 6, 7 |
| 37. Which insect species have been found on human remains? | 2 |
| 38. What are potential natural controls of potential pests of crop X? | 2 |
| 39. What are the potential vectors of crop disease X? | 2 |
| 40. Which species of the order X parasitize mammals in Africa? | 1, 2 |
| 41. Does species *X* make a cocoon? | 4, 5 |
| 42. What do we know about the development of species *X*? | 4 |
| 43. Is there developmental information about species *X* from lab rearings? | 4 |
| 44. What is known about the development time of species *X*? | 4 |
| 45. Which species of genus *X* are found in tropical lowland forests? | 6 |
| 46. Which insect genera are found in both forests and deserts? | 6 |
| 47. Which species are found in biome X? | 6 |
| 48. Under what temperature regimes do fireflies signal? | 3, 8 |
| 49. Which species in family X are nocturnal? | 1, 9 |
| 50. Has species *X* been collected at lights? | 9 |
| 51. Which non-target species are collected in pheromone traps for species *X*? | 9 |
| 52. If I want to observe/collect species *X*, what methods should I use? | 2, 6-9 |

**Table S2.** Ontology user domains or groups, example use cases, and the most relevant competency questions for each use case. Competency question numbers correspond with the question numbers in Table S1.

| User domain/group | Example use cases | Relevant competency questions |
|---|---|---|
| Entomology | Developmental studies | 1, 41-44, 52 |
| | Forensic entomology | 23, 26, 37, 42 |
| | Insect collecting and rearing | 1, 2, 8-13, 16-19, 22, 24, 26, 29-32, 40, 42-45, 47, 49-52 |
| | Medical and veterinary entomology | 18, 33-36, 40 |
| | Natural history studies | 1, 2, 4, 5, 8-13, 15-32, 40-50, 52 |
| Taxonomy and systematics | Authoring field guides | 1, 2, 8, 9, 12, 16, 18, 22, 23, 26, 29, 30, 40, 41, 45, 49 |
| | Generating checklists/catalogues | 1, 2, 9, 12, 16, 18, 22, 23, 26, 29, 30, 41, 45, 47, 49 |
| | Systematic revisions | 1, 2, 9, 12, 16, 18, 22, 23, 26, 29, 30, 41, 45, 49 |
| Ecology and evolutionary biology | Community ecology | 3-18, 20-23, 25, 26, 28-32, 40, 45, 47 |
| | Comparative analyses | 2, 8-12, 15-19, 21-23, 26, 28-32, 40, 41, 45-49 |
| | Disease ecology | 35, 39, 40 |
| | Ecosystem ecology | 3-5, 9, 10, 23, 25, 32, 45-47 |
| | Evolutionary ecology | 2, 8-12, 15-19, 21-23, 26, 28-32, 40, 41, 45-49 |
| Conservation biology and natural resource management | Ecological restoration | 1, 3, 5, 6, 12, 23, 29, 45-47 |
| | Environmental monitoring | 31, 45, 47-52 |
| | Habitat preservation decisions | 5-7, 9, 11, 12, 14, 23, 29, 45, 47 |
| | Predicting trophic/extinction cascades | 3-7, 12, 23, 25 |
| Agriculture and forestry | Finding potential biological control agents | 4, 15-19, 21-25, 32, 38 |
| | Finding potential disease vectors | 4, 18, 23, 24, 35, 36, 39, 40 |
| | Finding potential pest insects | 4, 12, 13, 18, 23, 24, 29, 35, 36, 38-40 |
| Education | Classroom education | 1-6, 8-12, 16-18, 20-27, 29-31, 33-37, 40-42, 45-49, 52 |
| | Public outreach | 1, 2, 6, 8-10, 12, 14, 17, 18, 22, 23, 27, 29, 30, 33-36, 38-40, 45, 47, 48, 52 |
| General public | Finding potential garden pests and control agents | 4, 8, 12, 16-19, 22-24, 29, 32, 38, 39 |
| | Insect collecting and rearing | 1, 2, 8-13, 16-19, 22, 24, 26, 29-32, 40, 42-45, 47, 49-52 |
| | Natural history studies | 1, 2, 4, 5, 8-13, 15-32, 40-50, 52 |
